# Covalently Tethering Atomically Precise Au_25_ Nanoclusters onto Covalent Organic Framework for Visible-Light-Driven CO_2_ Reduction

**DOI:** 10.34133/research.1152

**Published:** 2026-02-27

**Authors:** Ping Fu, Biao Meng, Qizhi Hu, Jiao Wei, Qihong Yue, Mingdong Sun, Qing Xu, Xiaoling Liu, Shibo Xi, Wen Yin, Yu Zhou, Jun Wang

**Affiliations:** ^1^ State Key Laboratory of Materials-Oriented Chemical Engineering, College of Chemical Engineering, Nanjing Tech University, Nanjing 211816, China.; ^2^ University of Chinese Academy of Sciences, Chinese Academy of Sciences, Beijing 101408, China.; ^3^ Spallation Neutron Source Science Center, Dongguan 523803, China.; ^4^ Institute of Sustainability for Chemicals, Energy and Environment, A*STAR (Agency for Science, Technology and Research), 1 Pesek Road, Jurong Island, Singapore 627833, Singapore.

## Abstract

Solar-driven carbon dioxide (CO_2_) reduction can sustainably produce chemicals and fuels but is often limited by rapid charge recombination and slow CO_2_-to-product kinetics, typically necessitating homogeneous photosensitizers and cocatalysts. Here, we reported an integrated photocatalyst, TF-COF-CONH-Au_25_-Co, constructed by immobilizing atomically precise Au_25_ nanoclusters (NCs) on a covalent organic framework (COF) incorporating [Co(bpy)_3_]^2+^ complex (Co–N_6_ coordination). Under visible-light illumination, this hybrid catalyzes CO_2_ conversion to syngas without external photosensitizers or cocatalysts, delivering a CO formation rate of 2,321.9 μmol·g^−1^·h^−1^ (turnover number of 171.9 and turnover frequency of 7.2 h^−1^). The Au_25_ NCs enhance light responsiveness and charge transfer efficiency, thereby enriching long-lived photogenerated electrons, while concurrently modulating the electronic state of Co sites to reduce the energy barrier for CO_2_ reduction. This study illustrates a molecular-level strategy to synergistically integrate metal NCs, COFs, and [Co(bpy)_3_]^2+^, showing a promising platform for high-performance photocatalytic CO_2_ conversion.

## Introduction

Solar-driven reduction of carbon dioxide (CO_2_) represents a compelling strategy for addressing both climate change and global energy challenges [1–4]. Nevertheless, the formidable CO_2_ thermodynamic stability and rapid charge recombination remain important obstacles to the development of efficient photocatalytic systems [5,6]. Homogeneous organometallic photosensitizers, typically Ru- or Re-based complexes, are normally involved in bridging the gap between sluggish CO_2_ reduction kinetics and the short lifetimes of excited states [3,5,7]. Nevertheless, their high price, limited durability, and recycling difficulties restrict their practical application [8], making the urgent need for photosensitizer-free heterogeneous CO_2_ photoreduction systems.

Atomically precise metal nanoclusters (NCs) are ligand-protected metal assemblies characteristic of well-defined chemical composition and structure [9–12]. Their subnanometer dimensions endow distinctive geometric and electronic properties, including abundant surface unsaturated sites, quantum confinement, and pronounced size effects [11,13–16]. In photocatalysis, the discrete energy level and the molecule-like behavior of metal NCs render excellent light harvesting across the ultraviolet–visible (UV–vis) near-infrared region [13,17,18]. They can function as narrow-bandgap semiconductors to generate photoexcited carriers and as the electron acceptors to suppress charge recombination [13,14,17]. However, their high surface free energy will easily lead to deactivation due to aggregation during the catalytic process [19,20]. Immobilization on well-defined porous supports via electrostatic interactions, encapsulation, coordination, or covalent bonding is therefore crucial to retain their structure and activity [20–24].

Covalent organic frameworks (COFs) are attractive supports due to the well-defined ordered porosity, tunable composition, ordered porosity, and predictable design [6,8,25–30]. Moreover, COF-based single-atom catalysts enable site-specific structural and functional control at the atomic level [8,31–34]. Here, we report the first example of immobilizing atomically precise Au_25_(Cys)_18_ NCs (Au_25_ NCs) on a COF scaffold bearing [Co(bpy)_3_]^2+^ complex. A 2-dimensional COF, named TF-COF, was synthesized via the condensation of 2,4,6-tris(4-formylphenyl)-1,3,5-triazine (TTA) and tris(4-formylphenyl)amine (TFA), followed by quinoline-4-carboxylic acid functionalization through an irreversible Doebner reaction to yield TF-COF-COOH [35,36]. Au_25_ NCs were covalently anchored through amidation between COF’s carboxyl groups and NCs’ amino groups, affording TF-COF-CONH-Au_25_. Subsequent incorporation of Co sites produced TF-COF-CONH-Au_25_-Co. The Au NCs promote the light absorption and act as electron sinks to prolong carrier lifetimes. Meanwhile, their strong electronic interaction with neighboring Co sites modulates the local environment, thereby reducing the activation energy of the key step in CO_2_ reduction. These synergistic effects enable TF-COF-CONH-Au_25_-Co to drive visible-light-mediated CO_2_ transformation into syngas, a valuable mixture of CO and H_2_ that has numerous industrial applications [32,37], without extra photosensitizers or cocatalysts.

## Results and Discussion

### Construction of Au_25_ NCs tethered to COF

**Fig. 1.**
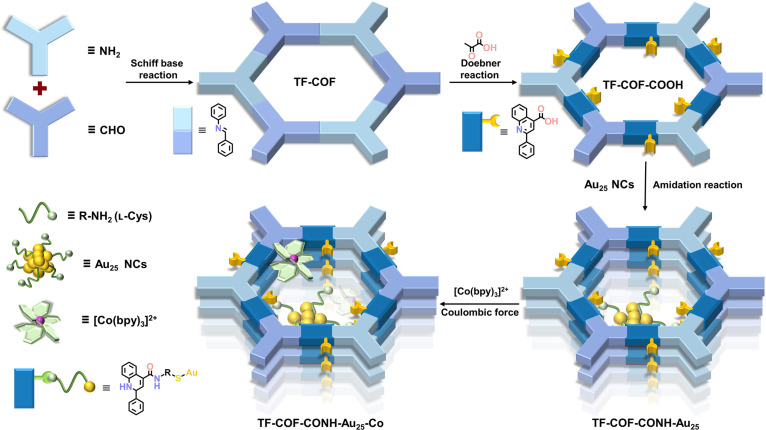
Synthesis schematic diagram. Schematic illustration showing the integration of Au_25_ NCs, COFs, and [Co(bpy)_3_]^2+^ complex.

**Fig. 2.**
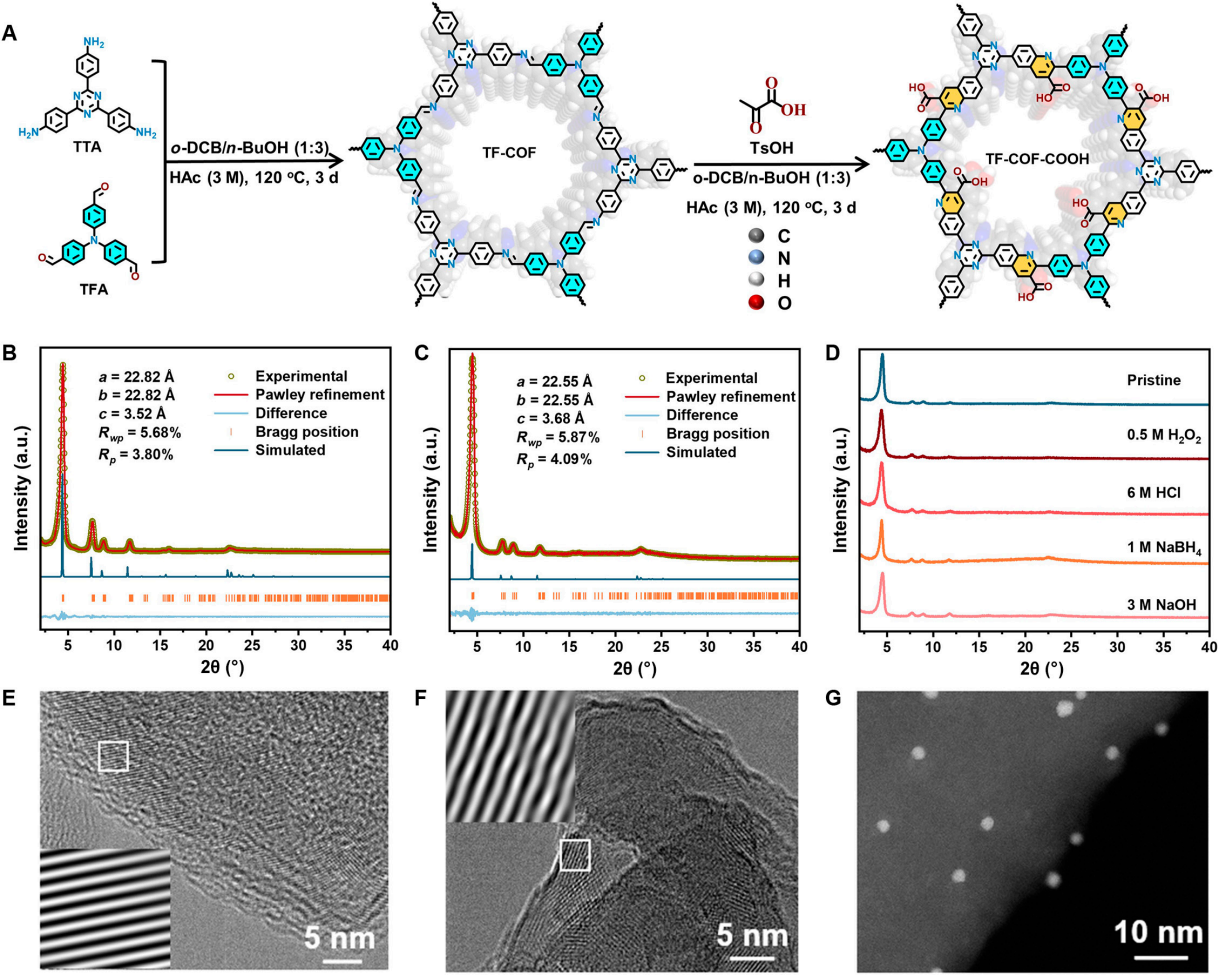
Crystal structure and morphology. (A) Synthetic procedure of TF-COF and TF-COF-COOH. *o*-DCB, *ortho*-dichlorobenzene; *n*-BuOH, *n*-butanol; HAc, acetic acid. PXRD patterns and Pawley refinements of (B) TF-COF and (C) TF-COF-COOH. a.u., arbitrary units. (D) PXRD patterns of TTA-TFA-COOH after treatment in 3 M NaOH, 6 M HCl, 1 M NaBH_4_, and 0.5 M H_2_O_2_ for 1 d. High-resolution TEM images of (E) TF-COF and (F) TF-COF-CONH-Au_25_. (G) HAADF-STEM image of TF-COF-CONH-Au_25_.

Figure 1 illustrated the overall strategy for the construction of Au_25_ NCs tethered to COFs, with the detailed synthetic route of the COF support shown in Fig. 2A. Au_25_ NCs were synthesized, and the structure was confirmed by UV–vis spectrum (Fig. S1) and high-resolution electrospray ionization mass spectrometry (Fig. S2) [20,38]. Powder x-ray diffraction (PXRD) analysis of TF-COF (Fig. 2B) displayed characteristic reflections at 4.39°, 7.66°, 8.88°, 11.74°, and 22.54°, indexing to the (100), (210), (200), (310), and (001) planes [39], respectively. TF-COF-COOH exhibited a nearly identical diffraction profile (Fig. 2C), indicating that the quinoline-4-carboxylic acid functionalization preserved the original COF framework. Simulated PXRD patterns based on an overlapping (AA) stacking model resembled the experimentally observed data (Fig. 2B and C and Figs. S3 and S4). Pawley refinements resulted in good agreement factors and provided the unit cell parameters (Fig. 2B and C). The chemical robustness of TF-COF-COOH was evaluated by exposure to harsh conditions, including strong base (3 M NaOH), strong acid (6 M HCl), oxidizing (0.5 M H_2_O_2_), and reducing (1 M NaBH_4_) environments for 24 h. PXRD patterns validated the retention of crystallinity (Fig. 2D), whereas TF-COF suffered framework collapse under the same treatments (Fig. S5), demonstrating that conversion of imine linkages to quinoline units enhanced chemical stability. Importantly, TF-COF-CONH-Au_25_ exhibited a PXRD pattern essentially identical to TF-COF-COOH (Fig. S6), verifying that covalent anchoring of Au_25_ NCs did not disrupt the COF lattice.

Fourier transform infrared (FT-IR) spectrum of TF-COF showed a characteristic band at 1,626 cm^−1^, assignable to the C═N stretching vibration of the imine unit (Fig. S7A) [40,41]. This band disappeared in TF-COF-COOH and TF-COF-CONH-Au_25_, accompanied by new absorptions at 1,722 and 1,608 cm^−1^, attributable to the C═O stretching of the quinoline-4-carboxylic acid moiety and the pyridyl group vibration [35,36], respectively. The C═O peak in the spectrum of TF-COF-CONH-Au_25_ was red-shifted relative to TF-COF-COOH (Fig. S7B), indicative of the amide bond generation [42,43]. Solid-state ^13^C nuclear magnetic resonance (NMR) spectra confirmed the structural transformation (Figs. S8 to S10). TF-COF displayed a resonance at 157 parts per million (ppm) from imine carbon, which disappeared in TF-COF-COOH, alongside the emergence of a –COOH signal at 172 ppm [35,36]. TF-COF-CONH-Au_25_ showed a C═O resonance at 174 ppm from the amide linkage and an additional peak at 64 ppm assignable to the methylene group of the Au_25_ NC ligand (Fig. S10). Nitrogen sorption measurement revealed the type I isotherms for TF-COF and TF-COF-COOH (Fig. S11A) [44]. The surface areas were 1,510 and 1,215 m^2^·g^−1^, respectively, with the most probable pore sizes of 21 Å (Fig. S11B and Table S1). TF-COF-CONH-Au_25_ exhibited a type IV isotherm with a reduced surface area of 640 m^2^·g^−1^, consistent with partial pore occupancy by bulky Au_25_ NCs. CO_2_ sorption isotherms at 298 K demonstrated that the uptake at 1 bar increased from 36.7 cm^3^·g^−1^ for TF-COF to 48.6 and 41.4 cm^3^·g^−1^, respectively, for TF-COF-COOH and TF-COF-CONH-Au_25_ (Fig. S12). The Au content in TF-COF-CONH-Au_25_ was approximately 5.56 wt %. Scanning electron microscopy (SEM) images of these COFs showed similar coral-bar morphology (Fig. S13), while elemental imaging confirmed homogeneous distribution of C, N, O, S, and Au in TF-COF-CONH-Au_25_ (Fig. S14). Transmission electron microscopy (TEM) images of TF-COF and TF-COF-COOH revealed well-resolved lattice fringes, indicative of high crystallinity (Fig. 2E and F). High-angle annular dark-field scanning TEM (HAADF-STEM) and TEM images of TF-COF-CONH-Au_25_ visualized uniformly distributed NCs (Fig. 2G and Fig. S15), confirming the incorporation of Au_25_ NCs.

X-ray photoelectron spectroscopy (XPS) was conducted to elucidate the surface chemical states (Figs. S16 to S20). The survey scan XPS spectra revealed the C 1s, N 1s, and O 1s signals at 291.4, 404.4, and 540.1 eV, respectively, while TF-COF-CONH-Au_25_ exhibited additional Au 4f (87.0 eV) and S 2p (162.0 eV) peaks (Fig. S16) [20,42]. High-resolution N 1s XPS spectra (Fig. S17) corroborated the integration of quinoline-4-carboxylic acid units in both TF-COF-COOH and TF-COF-CONH-Au_25_, with a characteristic quinoline nitrogen signal at 400.2 eV, alongside contributions from sp^3^-N (399.3 eV) and imine/triazine nitrogen (C═N; 400.3 eV) [39]. TF-COF-CONH-Au_25_ exhibited an extra component at 401.7 eV from the amide nitrogen [20]. The C 1s and O 1s spectra (Figs. S18 and S19) of TF-COF-COOH and TF-COF-CONH-Au_25_ presented the peaks at 288.8 and 532.4 eV, respectively, arising from the –COOH moieties [35,36]. The Au 4f and S 2p peaks of TF-COF-CONH-Au_25_ showed a positive binding energy shift compared to pristine Au_25_ NCs (Fig. S20), suggesting reduced electron densities on Au and S atoms due to the NC–COF interaction [20].

**Fig. 3.**
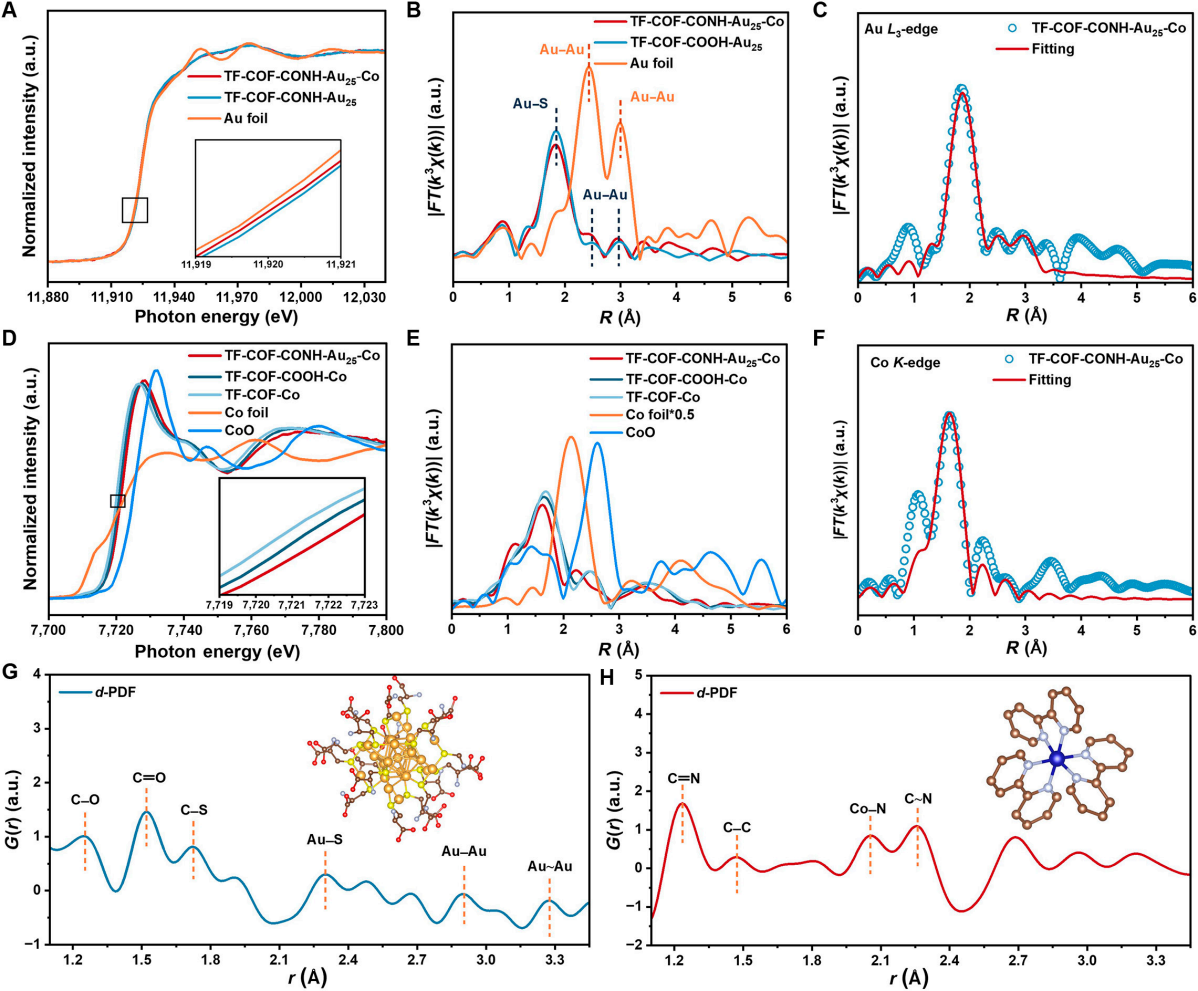
EXAFS spectra and neutral scattering. (A) XANES and (B) FT-EXAFS spectra at the Au *L*_3_-edge of TF-COF-CONH-Au_25_, TF-COF-CONH-Au_25_-Co, and Au foil. (C) EXAFS fitting curves at the Au *L*_3_-edge of TF-COF-CONH-Au_25_-Co. (D) XANES and (E) FT-EXAFS spectra at the Co *K*-edge of TF-COF-CONH-Au_25_-Co, TF-COF-COOH-Co, TF-COF-Co, Co foil, and CoO. (F) EXAFS fitting curves at the Co *K*-edge of TF-COF-CONH-Au_25_-Co. (G) *d*-PDF profile between TF-COF-CONH-Au_25_ and TF-COF-COOH. (H) *d*-PDF between TF-COF-CONH-Au_25_-Co and TF-COF-CONH-Au_25_. Blue data points and solid lines in (C) and (F) represent the experimental values and fitting values for TF-COF-CONH-Au_25_-Co, respectively.

The local metal coordination environment was measured by x-ray absorption fine structure (XAFS) analysis (Fig. 3A to F). The Au *L*_3_-edge x-ray absorption near-edge structure (XANES) spectrum of TF-COF-CONH-Au_25_ displayed a rising edge of TF-COF-CONH-Au_25_ close to that of Au foil (Fig. 3A), indicating that the Au_25_ NCs in TF-COF-CONH-Au_25_ retained a metallic state with a slight positive charge [45]. Fourier-transformed *L*_3_-weighted extended XAFS (FT-EXAFS) spectrum of TF-COF-CONH-Au_25_ (Fig. 3B) demonstrated a dominant peak at 1.8 Å for Au–S coordination in the outermost layer of Au_25_ NCs [46]. Two weak peaks were observed at 2.5 and 3.0 Å for Au–Au contribution in the inner core of Au_25_ NCs [46,47]. Multishell EXAFS fitting (Fig. 3C, Figs. S21 to S23, and Table S2) displayed Au–S coordination number (CN) of 1.6 at 2.3 Å and Au–Au CNs of 1.5 (2.8 Å), 1.6 (3.1 Å), and 1.9 (3.3 Å) in TF-COF-CONH-Au_25_, consistent with reported structures of Au_25_ NCs [48,49].

Neutron total scattering combined with pair distribution function (PDF) analysis was performed on TF-COF-COOH and TF-COF-CONH-Au_25_ to gain atomic-level structure insights [32,50]. Covalent bonding of Au_25_ NCs intensified several peaks and introduced new features in the PDF profiles (Fig. 3G and Figs. S24 to S26). Differential neutron PDF (*d*-PDF) was obtained by subtracting the neutron PDF of TF-COF-COOH from TF-COF-CONH-Au_25_, showing the correlations at *r* = 1.3, 1.5, 1.7, 2.3, 2.9, and 3.3 Å for C−O, C═C, C═S, Au−S, Au−Au, and Au~Au (inner core Au_13_ to outer Au atoms), respectively. The experimentally obtained *d*-PDF profile agreed with the refined one (Figs. S25 and S26), confirming the covalent tethering and structural integrity of the Au_25_ NCs within the COF frameworks.

### Anchoring of [Co(bpy)_3_]^2+^ complex

Co species were anchored by complexing the COFs with [Co(bpy)_3_]^2+^ cations via Coulombic interaction and pore confinement (Fig. 1) [51,52]. Zeta potential tests revealed that the potentials of TF-COF, TF-COF-COOH, and TF-COF-CONH-Au_25_ were negatively charged, while [Co(bpy)_3_]^2+^ exhibited a positive potential of +18.2 mV (Fig. S27), enabling their assembly through electrostatic attraction. The resulting TF-COF-CONH-Au_25_-Co had a Co loading of 1.91 wt %, while TF-COF-Co and TF-COF-COOH-Co exhibited a Co content of 1.76 and 2.13 wt %, respectively. PXRD, FT-IR, nitrogen sorption, and SEM analyses verified the preservation of crystalline structure, porosity, and morphology (Figs. S28 to S31). TEM image verified the existence of Au NCs (Fig. S32).

Survey scan XPS spectra revealed the Au and Co signals (Fig. S33). High-resolution Au 4f XPS spectra showed a negative shift of the Au 4f signals of TF-COF-CONH-Au_25_-Co relative to TF-COF-CONH-Au_25_ (Fig. S34), reflecting the decrease in the valence state of Au species due to the interaction between Co species and Au_25_ NCs [17]. High-resolution Co 2p XPS spectrum of COF-CONH-Au_25_-Co displayed peaks at 797.4 and 781.4 eV, confirming Co(II) species (Fig. S35) [53]. The Co 2p signals of TF-COF-COOH-Co and TF-COF-CONH-Au_25_-Co were red-shifted relative to TF-COF-Co, revealing a higher Co oxidation state (Fig. S35). XANES analysis further supported these findings. The Au *L*_3_-edge profile of TF-COF-CONH-Au_25_-Co resembled that of TF-COF-CONH-Au_25_ (Fig. 3A), with a slight negative shift suggesting reduced Au valence [54], while the Au coordination environment remained unchanged, confirming the structural integrity of Au_25_ NCs. In the Co *K*-edge XANES (Fig. 3D), the absorption edges of TF-COF-Co, TF-COF-COOH-Co, and TF-COF-CONH-Au_25_-Co align closely with CoO, indicating Co valence near +2. The subtle edge shifts were attributable to ligand-field/covalency differences from bpy-N coordination. A weak pre-edge at ~7,707 eV further supported a distorted octahedral Co(II), consistent with the Co 2p XPS result [55,56]. The Co valence increased in the sequence of TF-COF-Co < TF-COF-COOH-Co < TF-COF-CONH-Au_25_-Co, reflecting enhanced Co–COF interactions promoted by –COOH groups and Au_25_ NCs (Figs. S36 and S37) [55,57,58]. FT-EXAFS spectra of these Co-loaded COFs exhibited the Co–N contribution at 1.6 Å (Fig. 3E). No distinct peak corresponding to the Co–Co coordination was observed at ~2.1 Å, reflecting the atomically dispersed Co species [59,60]. EXAFS fitting gave the CN of ~6 and bond length of 2.1 Å for Co–N contribution (Fig. 3F, Figs. S38 to S42, and Table S3). The *d*-PDF profiles of TF-COF-CONH-Au_25_ and TF-COF-CONH-Au_25_-Co showed the presence of correlations at *r* = 1.3, 1.5, 2.1, and 2.3 Å, respectively, for C═N, C−C, Co−N, and C~N (pyridyl C–N) of loaded [Co(bpy)_3_]^2+^. This result revealed the existence of immobilized [Co(bpy)_3_]^2+^, which is further confirmed by the good match between the refined *d*-PDF profile and the experimentally obtained one (Fig. 3H and Figs. S43 to S45).

### Photocatalytic CO_2_ reduction

**Fig. 4.**
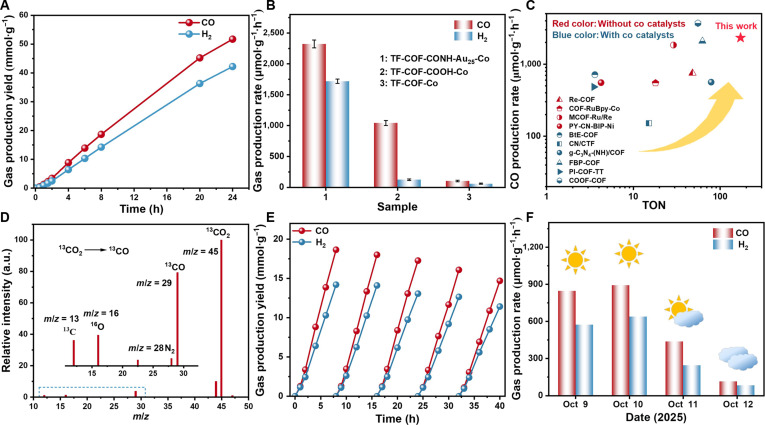
Photocatalytic performance. (A) Time-resolved generation of gas (CO and H_2_) over TF-COF-CONH-Au_25_-Co in 24 h. (B) Gas (CO and H_2_) production rates of TF-COF-Co, TF-COF-COOH-Co, and TF-COF-CONH-Au_25_-Co. (C) Comparison of TF-COF-CONH-Au_25_-Co with representative COF-based photocatalysts for CO_2_-to-CO conversion under visible-light irradiation without photosensitizers. The plot compares CO evolution rates and TON of reported photosensitizer-free COF systems, highlighting the superior performance of TF-COF-CONH-Au_25_-Co. (D) Mass spectrum of the gaseous product in TF-COF-CONH-Au_25_-Co-catalyzed ^13^CO_2_ conversion. (E) Recyclability of TF-COF-CONH-Au_25_-Co. (F) The photocatalytic CO production rate of TF-COF-CONH-Au_25_-Co under natural sunlight.

We performed the photocatalytic CO_2_ conversion under visible light irradiation (≥400 nm). TF-COF-CONH-Au_25_-Co demonstrated outstanding activity, continuously generating syngas (CO + H_2_) with linear product accumulation over time (Fig. 4A and Figs. S46 to S49). CO and H_2_ were produced at rates of 2,321.9 and 1,717.4 μmol·g^−1^·h^−1^, respectively, corresponding to the turnover number (TON) and turnover frequency (TOF) of 171.9 and 7.2 h^−1^ for CO generation. An ~1- to 2-h induction preceded steady state, arising from photoactivation of Co centers and triethanolamine (TEOA)-assisted charge separation build-up, as evidenced by the reversible growth of a 500- to 700-nm band in quasi in situ UV–vis spectra (Figs. S46 and S47) [61]. By contrast, TF-COF-COOH-Co achieved CO and H_2_ production rates of 1,042.7 and 124.8 μmol·g^−1^·h^−1^, respectively, while TF-COF-Co exhibited minimal activity (103.9 μmol·g^−1^·h^−1^ for CO and 60.4 μmol·g^−1^·h^−1^ for H_2_) (Fig. 4B). The superior performance of TF-COF-CONH-Au_25_-Co highlighted the synergy of Au_25_ NCs and Co centers. The inertness of Co-free analogs (TF-COF, TF-COF-COOH, and TF-COF-CONH-Au_25_) confirmed that Co species served as the active sites (Fig. S50). Au_25_ NCs produced no detectable CO and only trace H_2_ (76 μmol·g^−1^·h^−1^; Fig. S51), excluding Au_25_ as a carbon dioxide reduction reactioin (CO_2_RR) site. The physically mixed Au_25_+TF-COF-CONH-Co delivered a lower CO production rate of 1,313.0 μmol·g^−1^·h^−1^ (Fig. S51), underscoring that covalent tethering is critical for efficient interfacial charge transfer. The apparent quantum yield for TF-COF-CONH-Au_25_-Co reached 0.58% at 400 nm (Fig. S52). A comprehensive comparison with previous photosensitizer-free systems under comparable reaction conditions revealed that TF-COF-CONH-Au_25_-Co is advanced in terms of CO production rate, TON, and TOF (Fig. 4C and Table S4) [29,51,52,55,59,62].

Control experiments further verified the reaction pathway (Fig. S53). Certain H_2_ and trace CO were detected without the sacrificial agents. No product was detectable under dark conditions, illustrating the light dependence. Ar atmosphere led to the sole H_2_ production, suggesting the formation of CO from CO_2_. Photoreduction of ^13^CO_2_ (Fig. 4D) caused the formation of ^13^CO, as demonstrated by the ^13^CO signal (mass/charge ratio [*m*/*z*] = 29) in gas chromatography–mass spectrometry, substantiating CO_2_ as the carbon source [63]. ^1^H NMR spectrum excluded the generation of potential liquid products (Fig. S54). Over 5 consecutive 8-h cycles (40 h total), COF-CONH-Au_25_-Co sustained a CO rate of 1,798.8 μmol·g^−1^·h^−1^ (~77% retention; Fig. 4E). Postreaction PXRD of the recovered catalyst showed intact COF reflections without peak broadening or shifts (Fig. S55), while XPS confirmed preservation of the Au^0^ 4f_7/2_ and Co^2+^ 2p_3/2_ features and the absence of new oxide signatures (Fig. S56). Inductively coupled plasma mass spectrometry showed the recovered catalyst retained 1.62 wt % Co (~85% of the starting 1.91 wt %), showing only minor leaching and quite robust immobilization of Co complex. By contrast, the physically mixed Au_25_+TF-COF-CONH-Co exhibited poorer activity retention (Fig. S57), indicating that covalent anchoring markedly enhances durability. In addition, we further evaluated the photocatalytic performance of TF-COF-CONH-Au_25_-Co under natural sunlight (Fig. 4F). The CO and H_2_ production rate depended on the weather conditions, exhibiting the highest activity (891.6 and 637.5 μmol·g^−1^·h^−1^ for CO and H_2_) on 2025 October 10, a sunny day.

### Photoelectrochemical properties

**Fig. 5.**
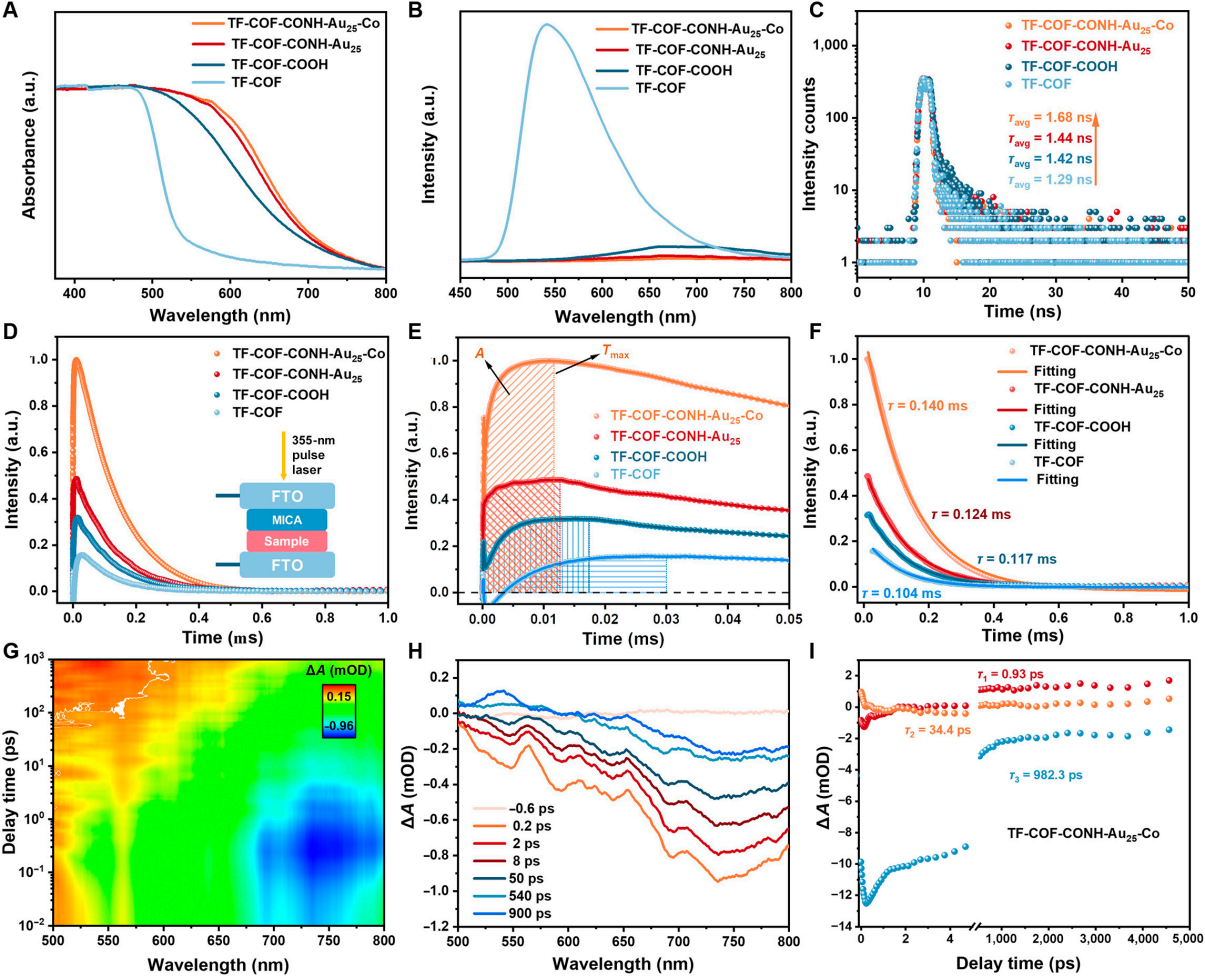
Photoelectrochemical properties. (A) UV–vis spectra, (B) PL spectra, (C) time-resolved PL decay spectra for TF-COF, TF-COF-COOH, TF-COF-CONH-Au_25_, and TF-COF-CONH-Au_25_-Co. (D) TPV curves, (E) maximum charge extraction time (*T*_max_), and (F) attenuation constants (*τ*) for TF-COF, TF-COF-COOH, TF-COF-CONH-Au_25_, and TF-COF-CONH-Au_25_-Co. FTO, fluorine-doped tin oxide. Light blue, dark blue, red, and orange data points and solid lines in (F) represent the experimental and fitting values for TF-COF, TF-COF-COOH, TF-COF-CONH-Au_25_, and TF-COF-CONH-Au_25_-Co, respectively. (G) 2D mapping TA spectra and (H) fs-TA spectra of TF-COF-CONH-Au_25_-Co at different decay times. (I) Representative kinetic traces along with their global fitting results of the fs-TA spectra of TF-COF-CONH-Au_25_-Co. mOD, milliopital density.

We systematically evaluated the photoelectrochemical properties of TF-COF, TF-COF-COOH, TF-COF-CONH-Au_25_, and their Co-loaded analogs. UV–vis spectra revealed that the introduction of 4-carboxyquinoline units into TF-COF induced a red shift in the absorption edge (Fig. 5A), attributed to enhanced π-conjugation [35,62]. The optical bandgap energy (*E*_g_) changed from 2.38 eV for TF-COF to 1.95 eV for TF-COF-COOH and 1.86 eV for TF-COF-CONH-Au_25_ (Figs. S58 and S59). Mott–Schottky analysis indicated n-type semiconducting behavior for all 3 COFs, with conduction band (CB) potentials of −0.86, −0.99, and −1.12 V versus the normal hydrogen electrode (NHE) [64], sufficiently negative for CO_2_-to-CO transformation (−0.53 V versus NHE) (Figs. S60 to S63) [32,40]. Co loading resulted in little effect on the band alignment but slightly enhanced visible-light absorption (Fig. 5A and Fig. S59).

Steady-state photoluminescence (PL) spectra showed a pronounced emission for TF-COF, while TF-COF-COOH, TF-COF-CONH-Au_25_, and TF-COF-CONH-Au_25_-Co demonstrated progressively quenched signals, reflecting the inhibited radiative recombination (Fig. 5B) [62]. Time-resolved PL spectra revealed prolonged carrier lifetimes by introducing –COOH (1.42 ns) and Au_25_ NCs (1.44 ns) relative to TF-COF (1.29 ns), with the longest lifetime for TF-COF-CONH-Au_25_-Co (1.68 ns) (Fig. 5C and Table S5) [20,35,62]. Transient surface photovoltage (TPV) measurements showed an increasing trend in both signal intensity and accumulated charge (*A*) from TF-COF to TF-COF-CONH-Au_25_-Co, along with shorter charge extraction times (*T*_max_) and larger decay constants (*τ*), with *τ* increasing from 0.104 μs (TF-COF) to 1.140 μs (TF-COF-CONH-Au_25_-Co) (Fig. 5D to F and Table S6), suggesting a obviously reduced recombination rate [65,66]. Electrochemical impedance spectroscopy (EIS) spectra showed the smallest semicircle for TF-COF-CONH-Au_25-_Co, corresponding to the lowest charge transfer resistance (Fig. S64) [28,55,67]. The photocurrent density followed the order of TF-COF < TF-COF-COOH < TF-COF-CONH-Au_25_ < TF-COF-CONH-Au_25_-Co (Fig. S65), in agreement with the improved charge separation and transport efficiency [55,62].

The photoelectrochemical properties of the other 2 Co-loaded materials, TF-COF-Co and TF-COF-COOH-Co, were compared with each parent COF. Co loading enhanced the charge separation and transfer as shown by the declined peak intensity in the PL spectra, extended carrier decay lifetime in the time-resolved PL spectra, increased TPV peak intensity, elongated decay constants, declined charge transfer resistance, and boosted photocurrent response (Figs. S66 to S70 and Tables S5 and S6) [55,68,69]. All these measurements indicate that the cooperation of Au_25_ NCs, COFs, and Co sites enhances light harvesting, facilitates charge separation and transfer, and thereby provides an abundance of long-lived photoelectrons.

Femtosecond transient absorption (fs-TA) spectra were collected to investigate the electron transfer dynamics. All samples exhibited a broad negative bleaching signal, indicative of ground-state bleaching, suggesting the excitation of carriers leading to exciton formation (Fig. 5G and H and Figs. S71 to S73). As the probe time progressed, this negative bleaching signal gradually transitioned into an excited-state absorption (ESA) signal, indicating the occurrence of charge transfer processes. Notably, TF-COF-CONH-Au_25_-Co displayed a obviously stronger ESA signal than TF-COF-CONH-Au_25_, TF-COF-COOH, and TF-COF [70]. Global fits to the TA kinetics resolved biexponential decays for TF-COF, TF-COF-COOH, and TF-COF-CONH-Au_25_ and a triexponential response for TF-COF-CONH-Au_25_-Co (Fig. 4I, Figs. S74 to S76, and Table S7) [71]. We assigned the fast component (*τ*_1_) to electron diffusion/dissociation within the COF domain and the slower component (*τ*_2_) to trap-assisted recombination [56,72]. TF-COF showed *τ*_1_ = 3.99 ps (*k*_1_ ≈ 2.5 × 10^11^ s^−1^) and *τ*_2_ = 365.2 ps (*k*_2_ ≈ 2.7 × 10^9^ s^−1^), while carboxylation modestly lengthens both *τ*_1_ (4.89 ps) and *τ*_2_ (437.6 ps) over TF-COF-COOH, consistent with slightly improved carrier separation [33]. Incorporating Au_25_ further extended lifetimes (*τ*_1_ = 5.87 ps and *τ*_2_ = 518.9 ps), indicating efficient exciton harvesting by the cluster yet recombination that still dominates on the subnanosecond scale in the absence of Co. By contrast, TF-COF-CONH-Au_25_-Co exhibited *τ*_1_ = 0.93 ps (*k*_1_ ≈ 1.1 × 10^12^ s^−1^), *τ*_2_ = 34.3 ps (*k*_2_ ≈ 2.9 × 10^10^ s^−1^), and an emergent long-lived component *τ*_3_ = 982.3 ps (*k*_3_ ≈ 1.0 × 10^9^ s^−1^). We attributed these to ultrafast COF → Au_25_ exciton capture (*τ*_1_), rapid Au_25_ → Co electron relay and stabilization at the cobalt center (*τ*_2_), and a persistent charge-separated state (*τ*_3_) that suppresses recombination [72]. Thus, covalent integration of Au_25_ (electron sink) with Co sites transforms a 4- to 6-ps/300- to 500-ps exciton recombination sequence into subpicosecond harvesting, tens-of-picosecond interfacial transfer, and ~1-ns charge separation, quantitatively accounting for the stronger time-resolved PL quenching, reduced EIS semicircles/TPV shifts, and higher CO formation rates.

### In situ spectral and theoretical study

**Fig. 6.**
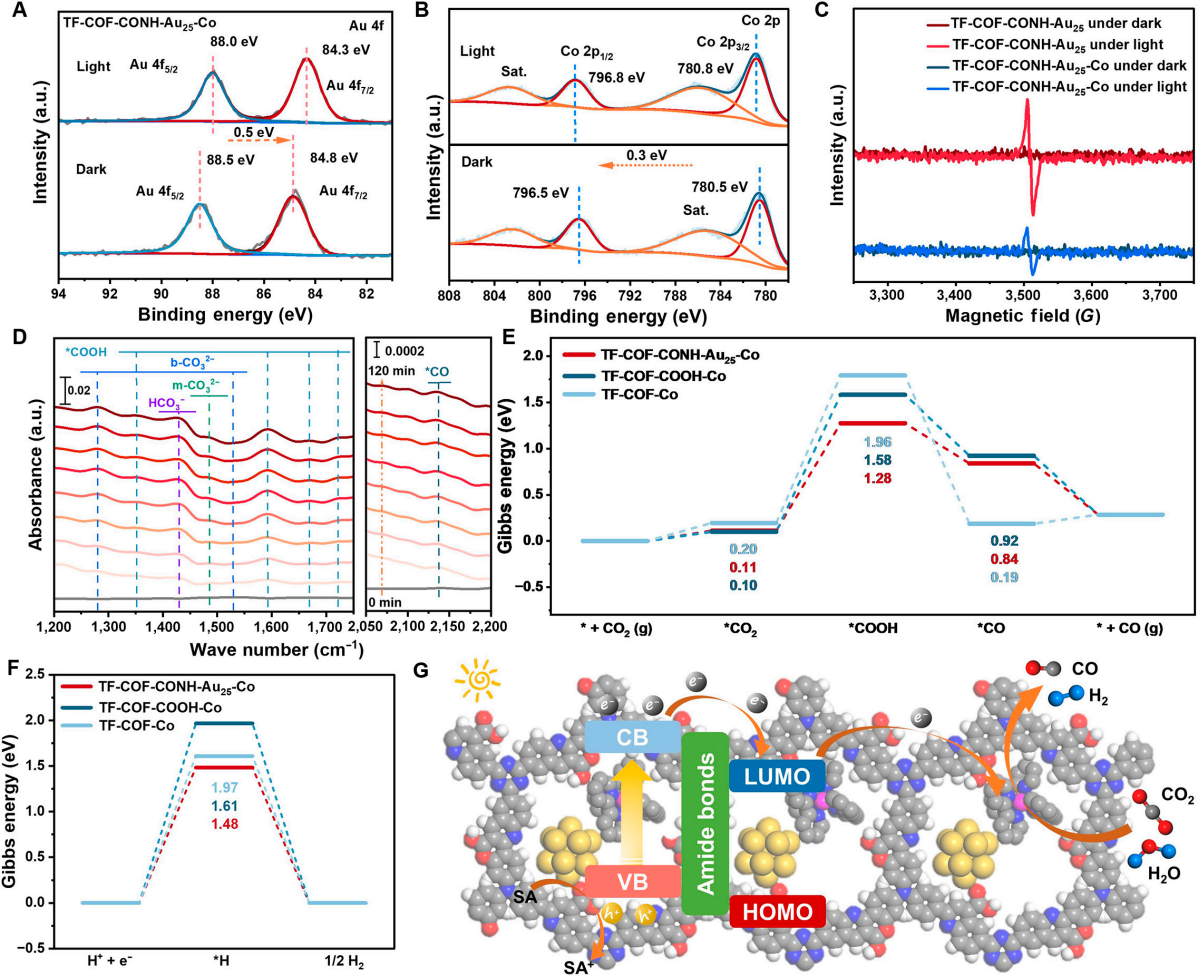
In situ spectral and theoretical study. Quasi in situ (A) Au 4f and (B) Co 2p XPS spectra of TF-COF-CONH-Au_25_-Co in the dark or under visible light illumination. (C) In situ EPR spectra of TF-COF-CONH-Au_25_ (red) and TF-COF-CONH-Au_25_-Co (blue) in MeCN/TEOA/BIH under dark and visible-light illumination. (D) In situ ATR-SEIRAS for the adsorption and photocatalytic conversion of CO_2_ on TF-COF-CONH-Au_25_-Co. Gibbs free energy profiles (Δ*G*, in electron volts) of (E) CO generation and (F) H_2_ evolution on TF-COF-Co, TF-COF-COOH-Co, and TF-COF-CONH-Au_25_-Co. (G) Photocatalytic CO_2_ conversion over TF-COF-CONH-Au_25_-Co. VB, valence band; HOMO, highest occupied molecular orbital; SA, sacrificial agent.

Quasi in situ XPS spectra of TF-COF-CONH-Au_25_-Co under light irradiation (Fig. 6A) indicated that the Au 4f level underwent a −0.5 eV shift, whereas the Co 2p level shifted by +0.3 eV (Fig. 6B). The negative shift on Au signifies transient electron accumulation, while the positive shift on Co indicates electron depletion (upward band bending), collectively evidencing a directional, light-driven COF → Au_25_ → Co electron-transfer cascade [73,74].

Photochromism further corroborated carrier generation and shuttling. Dispersions of TF-COF-CONH-Au_25_ in acetonitrile (MeCN)/TEOA/1,3-dimethyl-2-phenyl-2,3-dihydro-1*H*-benzo[d]imidazole (BIH) turned yellow to green under light. In situ electron paramagnetic resonance (EPR) revealed a strong growth of the paramagnetic signal after illumination (Fig. 6C). In contrast, the EPR intensity diminished when [Co(bpy)_3_]^2+^ is present (TF-COF-CONH-Au_25_-Co), indicating electron transfer from the COF/Au_25_ ensemble to the Co complex with the reduction of Co sites [75,76]. Cyclic voltammetry resolved 2 irreversible reductions assignable to [Co(bpy)_3_]^2+^ → [Co(bpy)_2_]^+^ and [Co(bpy)_2_]^+^ → [Co^I^(bpy)(bpy)^**•**−^]. Currents were enhanced under CO_2_ versus Ar, consistent with engagement of the reduced Co species in CO_2_ activation (Fig. S77) [55,56].

In situ attenuated total reflection surface enhanced infrared absorption spectroscopy (ATR-SEIRAS) measurements were used to track the CO_2_ photoreduction pathway over TF-COF-CONH-Au_25_-Co (Fig. 6D). Characteristic bands at 1,721 and 1,402 cm^−1^ were assignable to HCO_3_^−^, formed via coadsorption of CO_2_ and H_2_O [41]. Signals at 1,669, 1,593, and 1,352 cm^−1^ corresponded to the *COOH intermediate [77], while additional peaks at 1,559/1,281 cm^−1^ and 1,531/1,486 cm^−1^ indicated bidentate (b-CO_3_^2−^) [78] and monodentate (m-CO_3_^2−^) [37,79]. A distinct band at 2,134 cm^−1^ was assignable to *CO [34,79]. The gradual increase in peak intensities with irradiation time confirmed continuous formation of these intermediates, supporting a reaction sequence of CO_2_ adsorption, *COOH generation, *CO formation, and CO release. The Co-free control (TF-COF-CONH-Au_25_) showed only bicarbonate/carbonate signals (no *COOH/*CO; Fig. S78), whereas TF-COF-COOH-Co (no Au_25_) exhibited the same assignment but with lower *COOH/*CO intensities (Fig. S79). Together with EPR, these results establish Co complexes as the catalytic reduction sites and demonstrate that Au_25_ funnels electrons to Co, amplifying intermediate coverage and turnover.

Theoretical study using density functional theory (DFT) was conducted on representative frameworks of TF-COF-Co, TF-COF-COOH-Co, and TF-COF-CONH-Au_25_-Co (Fig. S80). As illustrated in Fig. 6E, the rate-determining step was the *COOH formation [34,80], with the barrier sequence of TF-COF-CONH-Au_25_-Co (1.17 eV) < TF-COF-COOH-Co (1.48 eV) < TF-COF-Co (1.76 eV). The lowest energy barrier of TF-COF-CONH-Au_25_-Co was thermodynamically responsive to its best activity. This sequence cohered with the more negative conduction band from Mott–Schottky analysis for TF-COF-CONH-Au_25_-Co, which provided a greater thermodynamic driving force for interfacial electron transfer, and with its longest TA carrier lifetime, indicating a larger steady-state electron population reaching the surface [81]. Together, these factors complemented the lowest computed kinetic barrier and rationalize the highest activity. Consistently, hydrogen evolution reaction (HER) energetics (Fig. 6F) revealed that TF-COF-CONH-Au_25_-Co has a smaller barrier for *H generation (1.48 eV) than TF-COF-Co (1.61 eV) and TF-COF-COOH-Co (1.97 eV), explaining its superior H_2_ evolution rate and syngas productivity.

Differential charge density and Bader charge analyses (Fig. S81) revealed that –COOH functionalization boosted electron transfer from the COF framework to the Co center, increasing from 0.65 e in TF-COF-Co to 0.94 e in TF-COF-COOH-Co [53,82]. Incorporation of Au_25_ NCs further increased electron transfer to 0.98 e, indicating that covalent Au–amide bonding strengthens Co–COF coupling and facilitates interfacial charge transport. Moreover, a near-linear correlation between Co 2p binding energy shifts and Bader charges (Fig. S82) placed TF-COF-CONH-Au_25_-Co at the largest positive shift/charge depletion, quantitatively evidencing strong Au_25_-induced electronic modulation at the Co center [22,83]. A mechanistic model is thus proposed in Fig. 6G. Under illumination, photogenerated holes are consumed by the sacrificial agent, while electrons migrate via the amide linkage to the lowest unoccupied molecular orbitals (LUMOs) of Au_25_ NCs, which act as electron reservoirs [74,84]. These electrons are subsequently transferred to the CO_2_-adsorbed [Co(bpy)_3_]^2+^ site through the COF framework (Fig. 6G), driving CO formation [55]. The synergistic effect of covalent anchoring and interfacial electrostatics not only stabilizes Au_25_ NCs but also optimizes the Co electronic environment, thereby lowering the barrier of the rate-limiting *COOH step and enhancing overall CO_2_ reduction efficiency.

## Conclusion

Atomically precise Au_25_ NCs were covalently immobilized on a carboxyl-functionalized COF via amide bond formation. Sequential incorporation [Co(bpy)_3_]^2+^ fabricated a highly efficient, photosensitizer-free heterogeneous catalyst for photocatalytic CO_2_ conversion into syngas under visible-light illumination. The immobilized Au NCs serve a dual function. First, they promote light-harvesting and accelerate charge separation and transfer by serving simultaneously as broadband absorbers and efficient electron sinks. Second, they adjust the electronic structure of nearby Co sites, optimizing intermediates binding and lowering the activation barrier of the rate-limiting step. This work presents a molecularly engineered photocatalyst that integrates [Co(bpy)_3_]^2+^, COFs, and atomically precise metal NCs into a unified platform, highlighting the great prospect of COFs as functional supports for metal NCs toward high-performance solar-to-chemical energy conversion.

## Materials and Methods

### Synthesis of TF-COF

To synthesize TF-COF, a 25-ml Pyrex tube was prepared with TTA (0.17 mmol, 67 mg), TFA (0.17 mmol, 56 mg), *n*-butanol (2.7 ml), *o*-dichlorobenzene (0.9 ml), and 3 M acetic acid (0.45 ml). The mixture was sonicated for 10 min and subsequently degassed at 77 K through 3 freeze–pump–thaw cycles. The tube was then sealed and heated at 120 °C for 3 d. The solid product formed was isolated using centrifugation, followed by successive washing with ethanol (3 × 10 ml) and tetrahydrofuran (THF; 3 × 10 ml). The resultant powder was dried under vacuum, yielding approximately 87% of yellow TF-COF.

### Synthesis of TF-COF-COOH

Similarly, a 25-ml Pyrex tube containing TF-COF (63 mg), *n*-butanol (2.7 ml), *o*-dichlorobenzene (0.9 ml), 3 M acetic acid (0.45 ml), *p*-toluenesulfonic acid (TsOH) (20 mg), and pyruvic acid (60 μl) was prepared. The mixture was sonicated for 10 min and degassed at 77 K through 3 freeze–pump–thaw cycles. After sealing, the mixture was heated at 120 °C for 3 d. The solid was collected via centrifugation and subjected to washing with ethanol (3 × 10 ml) and THF (3 × 10 ml). The final product, yellow TF-COF-COOH, was dried under vacuum, yielding an approximate 92% yield.

### Synthesis of TF-COF-CONH-Au_25_

To synthesize TF-COF-CONH-Au_25_, TF-COF-COOH (40 mg), *N*-hydroxysuccinimide (11.4 mg), and 1-ethyl-3-(3-dimethylaminopropyl)carbodiimide·HCl (37.8 mg) were combined in a 10-ml 2-(*n*-morpholino)ethanesulfonic acid (MES) solution (ethanol/H_2_O = 1/5, 4.2 mg·l^−1^) and stirred for 10 min. Subsequently, a 10-ml solution of Au_25_ NCs (0.57 mg·ml^−1^) was added to the mixture, which was then stirred for 2 h at room temperature. The resultant solid was isolated via centrifugation, washed with deionized water, and dried under vacuum to obtain the final product.

### Preparation of Co-loaded COF TF-COF-CONH-Au_25_-Co

To prepare the Co-loaded COF, CoCl_2_·6H_2_O (2 mg, 5.5 μmol) and 2,2′-bipyridine (4.3 mg, 27.5 μmol) were dissolved in 5 ml of MeCN. TF-COF-CONH-Au_25_ (10 mg) was then added to this solution. The mixture was stirred for 12 h under a nitrogen atmosphere at room temperature. Afterward, the solid was isolated through filtration, washed with water, and dried overnight in an oven at 60 °C to yield the Co-loaded product, TF-COF-CONH-Au_25_-Co. The same method was used for preparing TF-COF-Co and TF-COF-COOH-Co, utilizing the respective COF materials.

### Photocatalytic CO_2_ reduction

For the photocatalytic CO_2_ reduction, a gas-tight quartz reactor (90 ml) was filled with a powder sample (1 mg), MeCN (4 ml), TEOA (1 ml), and a solution of BIH (20 mg). High-purity CO_2_ (99.999 vol %) was then bubbled through the reaction mixture for 20 min to completely displace air. A 300-W xenon lamp (Microsolar 300, Beijing Perfectlight Technology Co. Ltd.) equipped with a UV cutoff filter (*λ* ≥ 400 nm) served as the light source to initiate the reaction at room temperature. The reaction setup was stirred vigorously using a magnetic stirrer throughout the photocatalytic process. After the reaction, gas samples were collected and analyzed using gas chromatography (GC-9860-5CNJ, Nanjing Hope Analytical Equipment Co. Ltd., China), using calibrations with standard gas mixtures. Hydrogen was detected using a thermal conductivity detector, while a flame ionization detector was utilized for measuring carbon monoxide and other hydrocarbons. The liquid phase products underwent identification through ^1^H NMR analysis. To evaluate the recycling potential of the catalyst, a 4-run recycling test was conducted, where, after each cycle, the catalyst was cleaned with water and subsequently isolated by centrifugation for the next cycle. To investigate the practical applicability, the catalytic efficiency of TF-COF-CONH-Au_25_-Co was evaluated in an open-air environment on the rooftop of a laboratory building from 2025 October 9 to 12.

### XAFS analysis

XAFS spectra were measured at the XAFCA beamline of Singapore Synchrotron Light Source with an electron energy of 0.7 GeV. Athena program was used for the energy calibration, background removal, and Fourier transform of the XAFS spectra. EXAFS fitting was conducted using Artemis software.

### Neutron total scattering

Neutron total scattering data were collected on a Multi-Physics Instrument at China Spallation Neutron Source (CSNS), Dongguan, China. The samples were pretreated under vacuum at 393 K for 3 h and then cooled to 10 K for the measurement. The total scattering *S*(*Q*) data were converted to PDF *G*(*r*) data through Fourier transformation. *d*-PDF spectrum was calculated from the PDF data of the COFs before and after Au_25_ NCs or [Co(bpy)_3_]^2+^ loading. PDF refinement was carried out on the program Topas-academic v.7. The structural models involved in the refinements were constructed according to the results of PXRD refinement, structure optimization by DFT calculation, and XANES simulation.

### In situ spectral and theoretical study

In situ ATR-SEIRAS spectra were collected on a Thermo Fisher Scientific Nicolet iS50 spectrometer (USA). DFT calculation was performed with the Vienna Ab initio Simulation Package. Experimental procedures for the in situ spectroscopy and computational details, together with other characterizations, are available in the Supplementary Materials.

## Data Availability

All data are available in the manuscript or Supplementary Materials or from the author.

## Supplementary Materials

Supplementary 1 Supplementary Text Figs. S1 to S82 Tables S1 to S7 References [85–112]

## References

[1] Fang S, Rahaman M, Bharti J, Reisner E, Robert M, Ozin GA, Hu YH. Photocatalytic CO_2_ reduction. Nat Rev Methods Primers. 2023;3(1):61.

[2] Xue Z-H, Luan D, Zhang H, Lou XW. Single-atom catalysts for photocatalytic energy conversion. Joule. 2022;6(1):92–133.

[3] Hu Y, Yu C, Wang S, Wang Q, Reinhard M, Zhang G, Zhan F, Wang H, Skoien D, Kroll T, et al. Identifying a highly efficient molecular photocatalytic CO_2_ reduction system via descriptor-based high-throughput screening. Nat Catal. 2025;8(2):126–136.

[4] Liu B, Wang T, Wang S, Zhang G, Zhong D, Yuan T, Dong H, Wu B, Gong J. Back-illuminated photoelectrochemical flow cell for efficient CO_2_ reduction. Nat Commun. 2022;13(1):7111. 36402767 10.1038/s41467-022-34926-x PMC9675791

[5] Nikoloudakis E, López-Duarte I, Charalambidis G, Ladomenou K, Ince M, Coutsolelos AG. Porphyrins and phthalocyanines as biomimetic tools for photocatalytic H_2_ production and CO_2_ reduction. Chem Soc Rev. 2022;51(16):6965–7045. 35686606 10.1039/d2cs00183g

[6] Lu M, Zhang M, Liu J, Chen Y, Liao JP, Yang MY, Cai YP, Li SL, Lan YQ. Covalent organic framework based functional materials: Important catalysts for efficient CO_2_ utilization. Angew Chem Int Ed Engl. 2022;61(15): Article e202200003. 35060268 10.1002/anie.202200003

[7] Kumagai H, Tamaki Y, Ishitani O. Photocatalytic systems for CO_2_ reduction: Metal-complex photocatalysts and their hybrids with photofunctional solid materials. Acc Chem Res. 2022;55(7):978–990. 35255207 10.1021/acs.accounts.1c00705 PMC8988296

[8] Mohata S, Majumder P, Banerjee R. Design and structure-function interplay in covalent organic frameworks for photocatalytic CO_2_ reduction. Chem Soc Rev. 2025;54:6062–6087. 40395047 10.1039/d5cs00106d

[9] Yao Q, Zhu M, Yang Z, Song X, Yuan X, Zhang Z, Hu W, Xie J. Molecule-like synthesis of ligand-protected metal nanoclusters. Nat Rev Mater. 2025;10(2):89–108.

[10] Li S, Li N-N, Dong X-Y, Zang S-Q, Mak TCW. Chemical flexibility of atomically precise metal clusters. Chem Rev. 2024;124(11):7262–7378. 38696258 10.1021/acs.chemrev.3c00896

[11] Jin R, Zeng C, Zhou M, Chen Y. Atomically precise colloidal metal nanoclusters and nanoparticles: Fundamentals and opportunities. Chem Rev. 2016;116(18):10346–10413. 27585252 10.1021/acs.chemrev.5b00703

[12] Ru HY, Yang JK, Yang YN, Wan QY, Zhu MJ, Hu JH, Li J, Li Q, Zhou M, Li G, et al. Unprecedented stacking-dependent piezoluminescence enhancement in atomically precise superatomic gold nanoclusters. Sci Adv. 2025;11(22):eadv0298. 40446027 10.1126/sciadv.adv0298 PMC12124367

[13] Li Y, Zhou M, Jin R. Programmable metal nanoclusters with atomic precision. Adv Mater. 2021;33(46):2006591. 10.1002/adma.202006591 33984169

[14] Yang D, Wang J, Wang Q, Yuan Z, Dai Y, Zhou C, Wan X, Zhang Q, Yang Y. Electrocatalytic CO_2_ reduction over atomically precise metal nanoclusters protected by organic ligands. ACS Nano. 2022;16(10):15681–15704. 36121680 10.1021/acsnano.2c06059

[15] Liu Z, Chen J, Li B, Jiang D-E, Wang L, Yao Q, Xie J. Enzyme-inspired ligand engineering of gold nanoclusters for electrocatalytic microenvironment manipulation. J Am Chem Soc. 2024;146(17):11773–11781. 38648616 10.1021/jacs.4c00019

[16] Qin Z, Li Z, Sharma S, Peng Y, Jin R, Li G. Self-assembly of silver clusters into one- and two-dimensional structures and highly selective methanol sensing. Research. 2022;2022: Article 0018. 39290962 10.34133/research.0018 PMC11407582

[17] Wang H, Zhang X, Zhang W, Zhou M, Jiang HL. Heteroatom-doped Ag_25_ nanoclusters encapsulated in metal-organic frameworks for photocatalytic hydrogen production. Angew Chem Int Ed Engl. 2024;63(17): Article e202401443. 38407530 10.1002/anie.202401443

[18] Sun QY, He HZ, Zhou Y, Dai YP, Shang P, Jiang XF. Photocatalytic hydroxylation and oxidative coupling reactions mediated by multinuclear Au(I) supramolecular clusters. Angew Chem Int Ed Engl. 2025;64(8): Article e202420499. 39715710 10.1002/anie.202420499

[19] Kollmannsberger KL, Kronthaler L, Jinschek JR, Fischer RA. Defined metal atom aggregates precisely incorporated into metal-organic frameworks. Chem Soc Rev. 2022;51(24):9933–9959. 36250400 10.1039/d1cs00992c

[20] Yao A, Du Y, Han M, Wang Y, Hu J, Zhu Q, Sheng H, Zhu M. Covalence bridge atomically precise metal nanocluster and metal-organic frameworks for enhanced photostability and photocatalysis. Nano Res. 2022;16(1):1527–1532.

[21] Huang R-W, Wei Y-S, Dong X-Y, Wu X-H, Du C-X, Zang S-Q, Mak TCW. Hypersensitive dual-function luminescence switching of a silver-chalcogenolate cluster-based metal-organic framework. Nat Chem. 2017;9(7):689–697. 28644463 10.1038/nchem.2718

[22] Wang H, Liu X, Yang W, Mao G, Meng Z, Wu Z, Jiang H-L. Surface-clean Au_25_ nanoclusters in modulated microenvironment enabled by metal-organic frameworks for enhanced catalysis. J Am Chem Soc. 2022;144(48):22008–22017. 36410048 10.1021/jacs.2c09136

[23] Cai X, Tian Y, Wang H, Huang S, Liu X, Li G, Ding W, Zhu Y. Catalytic N-formylation of CO_2_ by atomically precise Au_8_Pd_1_(DPPF)_4_^2+^ clusters into a two-dimensional metal-organic framework. Angew Chem Int Ed Engl. 2024;64(1): Article e202414030. 39267329 10.1002/anie.202414030

[24] Wang X, Zhao J, Eliasson H, Erni R, Ziarati A, McKeown Walker S, Bürgi T. Very low temperature CO oxidation over atomically precise Au_25_ nanoclusters on MnO_2_. J Am Chem Soc. 2023;145(50):27273–27281. 38065568 10.1021/jacs.3c06372 PMC10739995

[25] Ma T, Kapustin EA, Yin SX, Liang L, Zhou Z, Niu J, Li LH, Wang Y, Su J, Li J, et al. Single-crystal x-ray diffraction structures of covalent organic frameworks. Science. 2018;361(6397):48–52. 29976818 10.1126/science.aat7679

[26] Guan Q, Zhou LL, Dong YB. Metalated covalent organic frameworks: From synthetic strategies to diverse applications. Chem Soc Rev. 2022;51(15):6307–6416. 35766373 10.1039/d1cs00983d

[27] Liu R, Chen Y, Yu H, Položij M, Guo Y, Sum TC, Heine T, Jiang D. Linkage-engineered donor-acceptor covalent organic frameworks for optimal photosynthesis of hydrogen peroxide from water and air. Nat Catal. 2024;7(2):195–206.

[28] Huang Y, Du P, Shi WX, Wang Y, Yao S, Zhang ZM, Lu TB, Lu X. Filling COFs with bimetallic nanoclusters for CO_2_-to-alcohols conversion with H_2_O oxidation. Appl Catal B. 2021;288: Article 120001.

[29] Zou L, Chen ZA, Si DH, Yang SL, Gao WQ, Wang K, Huang YB, Cao R. Boosting CO_2_ photoreduction via regulating charge transfer ability in a one-dimensional covalent organic framework. Angew Chem Int Ed Engl. 2023;62(46): Article e202309820. 37768737 10.1002/anie.202309820

[30] Chen H, Jena HS, Feng X, Leus K, Van Der Voort P. Engineering covalent organic frameworks as heterogeneous photocatalysts for organic transformations. Angew Chem Int Ed Engl. 2022;61(47): Article e202204938. 36100584 10.1002/anie.202204938

[31] Chen L, Chen G, Gong C, Zhang Y, Xing Z, Li J, Xu G, Li G, Peng Y. Low-valence platinum single atoms in sulfur-containing covalent organic frameworks for photocatalytic hydrogen evolution. Nat Commun. 2024;15(1):10501. 39627232 10.1038/s41467-024-54959-8 PMC11614902

[32] Fu P, Chen C, Wu C, Meng B, Yue Q, Chen T, Yin W, Chi X, Yu X, Li R, et al. Covalent organic framework stabilized single CoN_4_Cl_2_ site boosts photocatalytic CO_2_ reduction into tunable syngas. Angew Chem Int Ed Engl. 2025;64(3): Article e202415202. 39193917 10.1002/anie.202415202

[33] Teng ZY, Zhang ZZ, Yang HB, Zhang QT, Ohno T, Su CL. Atomically isolated Sb(CN)_3_ on sp^2^-c-COFs with balanced hydrophilic and oleophilic sites for photocatalytic C-H activation. Sci Adv. 2024;10(5):eadl5432. 38295163 10.1126/sciadv.adl5432 PMC10830113

[34] Ran L, Li Z, Ran B, Cao J, Zhao Y, Shao T, Song Y, Leung MK, Sun L, Hou J. Engineering single-atom active sites on covalent organic frameworks for boosting CO_2_ Photoreduction. J Am Chem Soc. 2022;144(37):17097–17109. 36066387 10.1021/jacs.2c06920

[35] Das P, Chakraborty G, Roeser J, Vogl S, Rabeah J, Thomas A. Integrating Bifunctionality and chemical stability in covalent organic frameworks via one-pot multicomponent reactions for solar-driven H_2_O_2_ production. J Am Chem Soc. 2023;145(5):2975–2984. 36695541 10.1021/jacs.2c11454

[36] Yang Y, Yu L, Chu T, Niu H, Wang J, Cai Y. Constructing chemical stable 4-carboxyl-quinoline linked covalent organic frameworks via Doebner reaction for nanofiltration. Nat Commun. 2022;13(1):2615. 35550512 10.1038/s41467-022-30319-2 PMC9098490

[37] Lyu W, Liu Y, Chen D, Wang F, Li Y. Engineering the electron localization of metal sites on nanosheets assembled periodic macropores for CO_2_ photoreduction. Nat Commun. 2024;15(1):10589. 39632865 10.1038/s41467-024-54988-3 PMC11618665

[38] Hwang GB, Huang H, Wu G, Shin J, Kafizas A, Karu K, Toit HD, Alotaibi AM, Mohammad-Hadi L, Allan E, et al. Photobactericidal activity activated by thiolated gold nanoclusters at low flux levels of white light. Nat Commun. 2020;11(1):1207. 32139700 10.1038/s41467-020-15004-6 PMC7057968

[39] Xie Y, Pan T, Lei Q, Chen C, Dong X, Yuan Y, Maksoud WA, Zhao L, Cavallo L, Pinnau I, et al. Efficient and simultaneous capture of iodine and methyl iodide achieved by a covalent organic framework. Nat Commun. 2022;13(1):2878. 35610232 10.1038/s41467-022-30663-3 PMC9130143

[40] Lu M, Zhang M, Liu J, Yu TY, Chang JN, Shang LJ, Li SL, Lan YQ. Confining and highly dispersing single polyoxometalate clusters in covalent organic frameworks by covalent linkages for CO_2_ photoreduction. J Am Chem Soc. 2022;144(4):1861–1871. 35050618 10.1021/jacs.1c11987

[41] Zhou J, Li J, Kan L, Zhang L, Huang Q, Yan Y, Chen Y, Liu J, Li SL, Lan YQ. Linking oxidative and reductive clusters to prepare crystalline porous catalysts for photocatalytic CO_2_ reduction with H_2_O. Nat Commun. 2022;13(1):4681. 35948601 10.1038/s41467-022-32449-z PMC9365760

[42] Cui X, Wang J, Liu B, Ling S, Long R, Xiong Y. Turning Au nanoclusters catalytically active for visible-light-driven CO_2_ reduction through bridging ligands. J Am Chem Soc. 2018;140(48):16514–16520. 30407807 10.1021/jacs.8b06723

[43] Zhuo S, Wang X, Li L, Yang S, Ji Y. Chiral carboxyl-functionalized covalent organic framework for enantioselective adsorption of amino acids. ACS Appl Mater Interfaces. 2021;13(26):31059–31065. 34169712 10.1021/acsami.1c09238

[44] Li G, Yue Q, Fu P, Wang K, Zhou Y, Wang J. Ionic dye based covalent organic frameworks for photothermal water evaporation. Adv Funct Mater. 2023;33(34):2213810.

[45] Phongamwong T, Barrabés N, Donphai W, Witoon T, Rupprechter G, Chareonpanich M. Chlorophyll-modified Au_25_(SR)_18_-functionalized TiO_2_ for photocatalytic degradation of rhodamine B. Appl Catal B. 2023;325: Article 122336.

[46] Chen C, Ye C, Zhao X, Zhang Y, Li R, Zhang Q, Zhang H, Wu Y. Supported Au single atoms and nanoparticles on MoS_2_ for highly selective CO_2_-to-CH_3_COOH photoreduction. Nat Commun. 2024;15(1):7825. 39244601 10.1038/s41467-024-52291-9 PMC11380681

[47] Yu J, Gao RT, Guo X, Truong Nguyen N, Wu L, Wang L. Electrochemical nitrate reduction to ammonia on AuCu single-atom alloy aerogels under wide potential window. Angew Chem Int Ed Engl. 2025;64(4): Article e202415975. 39264141 10.1002/anie.202415975

[48] Shivhare A, Lee KE, Hu Y, Scott RWJ. Following the reactivity of Au_25_(SC_8_H_9_)_18_^−^ clusters with Pd^2+^ and Ag^+^ ions using in situ x-ray absorption spectroscopy: A tale of two metals. J Phys Chem C. 2015;119(40):23279–23284.

[49] Wang H, Liu X, Zhao Y, Sun Z, Lin Y, Yao T, Jiang H-L. Regulating interaction with surface ligands on Au_25_ nanoclusters by multivariate metal-organic framework hosts for boosting catalysis. Natl Sci Rev. 2024;11(10): Article nwae252. 39301064 10.1093/nsr/nwae252 PMC11409874

[50] Romero-Muñiz I, Mavrandonakis A, Albacete P, Vega A, Briois V, Zamora F, Platero-Prats AE. Unveiling the local structure of palladium loaded into imine-linked layered covalent organic frameworks for cross-coupling catalysis. Angew Chem Int Ed Engl. 2020;59(31):13013–13020. 32333630 10.1002/anie.202004197

[51] He Y, Zhao Y, Wang X, Liu Z, Yu Y, Li L. Multiple heteroatom-hydrogen bonds bridging electron transport in covalent organic framework-based supramolecular system for photoreduction of CO_2_. Angew Chem Int Ed Engl. 2023;62(31): Article e202307160. 37280761 10.1002/anie.202307160

[52] Zhou M, Wang Z, Mei A, Yang Z, Chen W, Ou S, Wang S, Chen K, Reiss P, Qi K, et al. Photocatalytic CO_2_ reduction using La-Ni bimetallic sites within a covalent organic framework. Nat Commun. 2023;14(1):2473. 37120625 10.1038/s41467-023-37545-2 PMC10148855

[53] Deng A, Zhao E, Li Q, Sun Y, Liu Y, Yang S, He H, Xu Y, Zhao W, Song H, et al. Atomic cobalt-silver dual-metal sites confined on carbon nitride with synergistic Ag nanoparticles for enhanced CO_2_ photoreduction. ACS Nano. 2023;17(12):11869–11881. 37289089 10.1021/acsnano.3c03176

[54] Zhou L, Qu Z, Fu L, Ding Y. Interfacial engineering of In-SnO_2_ heterostructure for promoting electrocatalytic CO_2_ reduction to formate. Appl Catal B. 2025;377: Article 125471.

[55] Yu M, Chen W, Lin Q, Li L, Liu Z, Bi J, Yu Y. Electrostatic confinement-induced excited charge transfer in ionic covalent organic framework promoting CO_2_ reduction. Angew Chem Int Ed Engl. 2025;64(6): Article e202418422. 39492798 10.1002/anie.202418422

[56] Zeng R, Sun C, Lin Z, Li Y, Zhou C, Zhang S, Li L, Guo S. Biomimetic interface with dynamic disulfide bonds boosts durable photoconversion of diluted CO_2_. J Am Chem Soc. 2025;147(42):38311–38319. 41069271 10.1021/jacs.5c10865

[57] Li M, Zuo Z, Zhang S. High-density ultrafine Au nanocluster-doped Co-LDH nanocages for enhanced visible-light-driven CO_2_ reduction. ACS Catal. 2023;13(17):11815–11824.

[58] Tian J, Zhong K, Zhu X, Yang J, Mo Z, Liu J, Dai J, She Y, Song Y, Li H, et al. Highly exposed active sites of Au nanoclusters for photocatalytic CO_2_ reduction. Chem Eng J. 2023;451: Article 138392.

[59] Xiang Y, Dong W, Wang P, Wang S, Ding X, Ichihara F, Wang Z, Wada Y, Jin S, Weng Y, et al. Constructing electron delocalization channels in covalent organic frameworks powering CO_2_ photoreduction in water. Appl Catal B. 2020;274: Article 119096.

[60] Liang J, Zhang H, Song Q, Liu Z, Xia J, Yan B, Meng X, Jiang Z, Lou XW, Lee CS. Modulating charge separation of oxygen-doped boron nitride with isolated Co atoms for enhancing CO_2_-to-CO photoreduction. Adv Mater. 2024;36(1):2303287. 10.1002/adma.202303287 37973198

[61] Yang S, Hu W, Zhang X, He P, Pattengale B, Liu C, Cendejas M, Hermans I, Zhang X, Zhang J, et al. 2D covalent organic frameworks as intrinsic photocatalysts for visible light-driven CO_2_ reduction. J Am Chem Soc. 2018;140(44):14614–14618. 30352504 10.1021/jacs.8b09705

[62] Lin H, Liu Y, Wang Z, Ling L, Huang H, Li Q, Cheng L, Li Y, Zhou J, Wu K, et al. Enhanced CO_2_ photoreduction through spontaneous charge separation in end-capping assembly of heterostructured covalent-organic frameworks. Angew Chem Int Ed Engl. 2022;61(50): Article e202214142. 36225162 10.1002/anie.202214142

[63] Lei B, Zhou G, Gong Z, Liu C, Zhou Y, Guro VP, Sun Y, Sheng J, Dong F. Dynamically cyclic Fe^2+^/Fe^3+^ active sites as electron and proton-feeding centers boosting CO_2_ photoreduction powered by benzyl alcohol oxidation. Research. 2024;8: Article 0567. 39801506 10.34133/research.0567 PMC11717996

[64] Yang Y, Zhang HY, Wang Y, Shao LH, Fang L, Dong H, Lu M, Dong LZ, Lan YQ, Zhang FM. Integrating enrichment, reduction, and oxidation sites in one system for artificial photosynthetic diluted CO_2_ reduction. Adv Mater. 2023;35(40):2304170. 10.1002/adma.202304170 37363880

[65] Yue Q, Zhang Z, Liu X, Zhu C, Wen Y, Fu P, Hu Q, Qu X, Zhou Y, Wang J. Engineering electron delocalization of ultrathin covalent organic framework nanosheets to elevate photocatalytic hydrogen evolution in seawater. Chem Eng J. 2025;507: Article 160481.

[66] Chen Q, Liu Y, Gu X, Li D, Zhang D, Zhang D, Huang H, Mao B, Kang Z, Shi W. Carbon dots mediated charge sinking effect for boosting hydrogen evolution in Cu-In-Zn-S QDs/MoS_2_ photocatalysts. Appl Catal B. 2022;301: Article 120755.

[67] Zhang LX, Qi MY, Tang ZR, Xu YJ. Heterostructure-engineered semiconductor quantum dots toward photocatalyzed-redox cooperative coupling reaction. Research. 2023;6: Article 0073. 36930756 10.34133/research.0073 PMC10013965

[68] Liu Y, Sun J, Huang H, Bai L, Zhao X, Qu B, Xiong L, Bai F, Tang J, Jing L. Improving CO_2_ photoconversion with ionic liquid and Co single atoms. Nat Commun. 2023;14(1):1457. 36928357 10.1038/s41467-023-36980-5 PMC10020152

[69] Yang S, Byun WJ, Zhao F, Chen D, Mao J, Zhang W, Peng J, Liu C, Pan Y, Hu J, et al. CO_2_ enrichment boosts highly selective infrared-light-driven CO_2_ conversion to CH_4_ by UiO-66/Co_9_S_8_ photocatalyst. Adv Mater. 2024;36(16):2312616. 10.1002/adma.202312616 38190551

[70] Cheng K, Kong S, Wang J, Wang Q, Yuan S, Li PZ, Zhao Y. Integrating multifunctionalities into a 3D covalent organic framework for efficient CO_2_ photoreduction. Angew Chem Int Ed Engl. 2025;64(26): Article e202504772. 40259635 10.1002/anie.202504772 PMC12184286

[71] Cao Y, Yu W, Li Y, Meng J, Zheng K, Huang C, Yang X, Yang Y, Dong F, Zhou Y. Engineering ultrafast photo-induced charge and carbon intermediates transfer at Interface to break the activity-selectivity trade-off in direct conversion of methane to methanol. Adv Energy Mater. 2024;15(6):2404871.

[72] Qiu J, Meng K, Zhang Y, Cheng B, Zhang J, Wang L, Yu J. COF/In_2_S_3_ S-scheme Photocatalyst with enhanced light absorption and H_2_O_2_-production activity and fs-TA investigation. Adv Mater. 2024;36(24):2400288. 10.1002/adma.202400288 38411357

[73] Yu Y, Dong XA, Chen P, Geng Q, Wang H, Li J, Zhou Y, Dong F. Synergistic effect of Cu single atoms and Au-Cu alloy nanoparticles on TiO_2_ for efficient CO_2_ photoreduction. ACS Nano. 2021;15(9):14453–14464. 34469113 10.1021/acsnano.1c03961

[74] Yan X, Fu XY, Xiao FX. Filling the gap: Atomically precise metal nanoclusters-induced Z-scheme photosystem toward robust and stable solar hydrogen generation. Adv Funct Mater. 2023;33(48):2303737.

[75] Chen X, Dang Q, Sa R, Li L, Li L, Bi J, Zhang Z, Long J, Yu Y, Zou Z. Integrating single Ni sites into biomimetic networks of covalent organic frameworks for selective photoreduction of CO_2_. Chem Sci. 2020;11(26):6915–6922. 33033603 10.1039/d0sc01747g PMC7499818

[76] Wang J, Xia T, Wang L, Zheng X, Qi Z, Gao C, Zhu J, Li Z, Xu H, Xiong Y. Enabling visible-light-driven selective CO_2_ reduction by doping quantum dots: Trapping electrons and suppressing H_2_ evolution. Angew Chem Int Ed Engl. 2018;57(50):16447–16451. 30350910 10.1002/anie.201810550

[77] Zhang AA, Wang ZX, Fang ZB, Li JL, Liu TF. Long-range π-π stacking brings high electron delocalization for enhanced photocatalytic activity in hydrogen-bonded organic framework. Angew Chem Int Ed Engl. 2024;63(46): Article e202412777. 39113321 10.1002/anie.202412777

[78] Wang J, Zhu W, Meng F, Bai G, Zhang Q, Lan X. Integrating dual-metal sites into covalent organic frameworks for enhanced photocatalytic CO_2_ reduction. ACS Catal. 2023;13(7):4316–4329.

[79] Su B, Wang S, Xing W, Liu K, Hung SF, Chen X, Fang Y, Zhang G, Zhang H, Wang X. Synergistic Ru species on poly(heptazine imide) enabling efficient photocatalytic CO_2_ reduction with H_2_O beyond 800 nm. Angew Chem Int Ed Engl. 2025;64(27): Article e202505453. 40275803 10.1002/anie.202505453

[80] Liu Z, Yin H, Sun J, Bai L, Li Z, Zhao X, Yan X, Zhao M, Jing L. Engineering single Cu atoms anchored via N-heterocyclic carbene in COF mesopores for modulating electron kinetics of CO_2_ photoconversion. Adv Energy Mater. 2024;14(33):2401713.

[81] Gu X, Niu H, Shi Y, Ding H, Cai Y, Jiang G. Cross-shaped donor-π-acceptor covalent organic frameworks with tunable push-pull architectures for effective photocatalytic H_2_O_2_ production. Angew Chem Int Ed Engl. 2025;65(5): Article e20491. 41363815 10.1002/anie.202520491

[82] Yu Y, He Y, Yan P, Wang S, Dong F. Boosted C-C coupling with Cu-Ag alloy sub-nanoclusters for CO_2_-to-C_2_H_4_ photosynthesis. Proc Natl Acad Sci USA. 2023;120(44): Article e2307320120. 37871220 10.1073/pnas.2307320120 PMC10622893

[83] Li T, Yang Z, Xu Q, Sun T, Ma X, Zheng T, Wang Y, Wang Z, Li Q, Yu Q, et al. Asymmetrical degree engineered carbon dioxide photoreduction for single atomic Co sites on polymeric carbon nitride. Adv Funct Mater. 2025;35(51): Article e11356.

[84] Ziarati A, Zhao J, Afshani J, Kazan R, Perez Mellor A, Rosspeintner A, McKeown S, Bürgi T. Advanced catalyst for CO_2_ photo-reduction: From controllable product selectivity by architecture engineering to improving charge transfer using stabilized Au clusters. Small. 2023;19(24): Article 2207857. 10.1002/smll.202207857 36895069

[85] Tan Y, Yan L, Huang C, Zhang W, Qi H, Kang L, Pan X, Zhong Y, Hu Y, Ding Y. Fabrication of an Au_25_-Cys-Mo electrocatalyst for efficient nitrogen reduction to ammonia under ambient conditions. Small. 2021;17(21):2100372. 10.1002/smll.202100372 33864356

[86] Wang C, Lv P, Xue D, Cai Y, Yan X, Xu L, Fang J, Yang Y. Zero-dimensional/two-dimensional Au_25_(Cys)_18_ nanoclusters/g-C_3_N_4_ nanosheets composites for enhanced photocatalytic hydrogen production under visible light. ACS Sustain Chem Eng. 2018;6(7):8447–8457.

[87] Lam E, Reisner E. A TiO_2_-Co(terpyridine)_2_ photocatalyst for the selective oxidation of cellulose to formate coupled to the reduction of CO_2_ to syngas. Angew Chem Int Ed Engl. 2021;60(43):23306–23312. 34464003 10.1002/anie.202108492

[88] Bonchio M, Bonin J, Ishitani O, Lu TB, Morikawa T, Morris AJ, Reisner E, Sarkar D, Toma FM, Robert M. Best practices for experiments and reporting in photocatalytic CO_2_ reduction. Nat Catal. 2023;6(8):657–665.

[89] Kudo A, Miseki Y. Heterogeneous photocatalyst materials for water splitting. Chem Soc Rev. 2009;38(1):253–278. 19088977 10.1039/b800489g

[90] Qureshi M, Takanabe K. Insights on measuring and reporting heterogeneous photocatalysis: Efficiency definitions and setup examples. Chem Mater. 2016;29(1):158–167.

[91] Kresse G, Furthmüller J. Efficient iterative schemes for ab initio total-energy calculations using a plane-wave basis set. Phys Rev B. 1996;54(16):11169. 10.1103/physrevb.54.11169 9984901

[92] Kresse G, Furthmüller J. Efficiency of ab-initio total energy calculations for metals and semiconductors using a plane-wave basis set. Comput Mater Sci. 1996;6(1):15–50.

[93] Perdew JP, Burke K, Ernzerhof M. Generalized gradient approximation made simple. Phys Rev Lett. 1996;77(18):3865–3868. 10062328 10.1103/PhysRevLett.77.3865

[94] Kresse G, Joubert D. From ultrasoft pseudopotentials to the projector augmented-wave method. Phys Rev B. 1999;59(3):1758.

[95] Blöchl PE. Projector augmented-wave method. Phys Rev B. 1994;50(24):17953. 10.1103/physrevb.50.17953 9976227

[96] Bhati M, Chen Y, Senftle TP. Density functional theory modeling of photo-electrochemical reactions on semiconductors: H_2_ evolution on 3C-SiC. J Phys Chem C. 2020;124(49):26625–26639.

[97] Gauthier JA, Ringe S, Dickens CF, Garza AJ, Bell AT, Head-Gordon M, Nørskov JK, Chan K. Challenges in modeling electrochemical reaction energetics with polarizable continuum models. ACS Catal. 2019;9(2):920–931.

[98] Mathew K, Sundararaman R, Letchworth-Weaver K, Arias TA, Hennig RG. Implicit solvation model for density-functional study of nanocrystal surfaces and reaction pathways. J Chem Phys. 2014;140(8): Article 084106. 24588147 10.1063/1.4865107

[99] Heaven MW, Dass A, White PS, Holt KM, Murray RW. Crystal structure of the gold nanoparticle [N(C_8_H_17_)_4_][Au_25_(SCH_2_CH_2_Ph)_18_]. J Am Chem Soc. 2008;130(12):3754–3755. 18321116 10.1021/ja800561b

[100] Dainese T, Antonello S, Gascon JA, Pan F, Perera NV, Ruzzi M, Venzo A, Zoleo A, Rissanen K, Maran F. Au_25_(SEt)_18_, a nearly naked thiolate-protected Au_25_ cluster: Structural analysis by single crystal x-ray crystallography and electron nuclear double resonance. ACS Nano. 2014;8(4):3904–3912. 24628268 10.1021/nn500805n

[101] Xia YS, Tang M, Zhang L, Liu J, Jiang C, Gao GK, Dong LZ, Xie LG, Lan YQ. Tandem utilization of CO_2_ photoreduction products for the carbonylation of aryl iodides. Nat Commun. 2022;13(1):2964. 35618727 10.1038/s41467-022-30676-y PMC9135707

[102] Tu W, Yang Y, Chen C, Zhou T, Li T, Wang H, Wu S, Zhou Y, O’Hare D, Zou Z, et al. Cu-O/N single sites incorporated 2D covalent organic framework ultrathin nanobelts for highly selective visible-light-driven CO_2_ reduction to CO. Small Struct. 2022;4(6):2200233.

[103] Dong P, Xu X, Luo R, Yuan S, Zhou J, Lei J. Postsynthetic annulation of three-dimensional covalent organic frameworks for boosting CO_2_ photoreduction. J Am Chem Soc. 2023;145(28):15473–15481. 37421363 10.1021/jacs.3c03897

[104] Gong LJ, Liu LY, Zhao SS, Yang SL, Si DH, Wu QJ, Wu Q, Huang YB, Cao R. Rapid charge transfer in covalent organic framework via through-bond for enhanced photocatalytic CO_2_ reduction. Chem Eng J. 2023;458: Article 141360.

[105] Zou L, Sa R, Zhong H, Lv H, Wang X, Wang R. Photoelectron transfer mediated by the interfacial electron effects for boosting visible-light-driven CO_2_ reduction. ACS Catal. 2022;12(6):3550–3557.

[106] Han WK, Li J, Zhu RM, Wei M, Xia SK, Fu JX, Zhang J, Pang H, Li MD, Gu ZG. Photosensitizing metal covalent organic framework with fast charge transfer dynamics for efficient CO_2_ photoreduction. Chem Sci. 2024;15(22):8422–8429. 38846403 10.1039/d4sc01896f PMC11151834

[107] Qin L, Sun D, Ma D, Wang Z, Liu Y, Li Q, Song F, Wu K, Gan L, Zhou T, et al. Decoupling interlayer interactions boosts charge separation in covalent organic frameworks for high-efficiency photocatalytic CO_2_ reduction. Adv Mater. 2025;37(29):2504205. 10.1002/adma.202504205 40297903

[108] Dong H, Fang L, Chen KX, Wei JX, Li JX, Qiao X, Wang Y, Zhang FM, Lan YQ. Dual metallosalen-based covalent organic frameworks for artificial photosynthetic diluted CO_2_ reduction. Angew Chem Int Ed Engl. 2025;64(2): Article e202414287. 39373554 10.1002/anie.202414287

[109] He J, Wang X, Jin S, Liu Z-Q, Zhu M. 2D metal-free heterostructure of covalent triazine framework/g-C_3_N_4_ for enhanced photocatalytic CO_2_ reduction with high selectivity. Chin J Catal. 2022;43(5):1306–1315.

[110] Wang J, Yu Y, Cui J, Li X, Zhang Y, Wang C, Yu X, Ye J. Defective g-C_3_N_4_/covalent organic framework van der Waals heterojunction toward highly efficient S-scheme CO_2_ photoreduction. Appl Catal B. 2022;301: Article 120814.

[111] Fu Z, Shu C, Wang X, Chen L, Wang X, Liu L, Wang K, Clowes R, Chong SY, Wu X, et al. Fluorinated covalent organic frameworks coupled with molecular cobalt cocatalysts for efficient photocatalytic CO_2_ reduction. CCS Chem. 2023;5(10):2290–2300.

[112] Kim YH, Jeon JP, Kim Y, Noh HJ, Seo JM, Kim J, Lee G, Baek JB. Cobalt-porphyrin-based covalent organic frameworks with donor-acceptor units as photocatalysts for carbon dioxide reduction. Angew Chem Int Ed Engl. 2023;62(36): Article e202307991. 37448236 10.1002/anie.202307991

