# Supplementary material for: Covalently Tethering Atomically Precise Au25 Nanoclusters onto Covalent Organic Framework for Visible-Light-Driven CO2 Reduction
Source: Research (Wash D C). 2026 Feb 27;9:1152. doi: 10.34133/research.1152 (PMC12946386; doi:10.34133/research.1152)
Supplement: Supplementary 1 — Supplementary Text Figs. S1 to S82 Tables S1 to S7 References [85–112] [file research.1152.f1.docx]

Supplemental Materials for

**Covalently Tethering Atomically Precise Au_25_ Nanoclusters onto Covalent Organic Framework for Visible-light Driven CO_2_ Reduction**

**This PDF file includes:**

Supplementary Text

Figs. S1 to S82

Tables S1 to S7

Supplementary Text

Materials and reagents

All reagents and solvents used in this study were of analytical grade and did not require further purification. N-butanol and *o*-dichlorobenzene were sourced from Energy Chemical, while cobalt(Ⅱ) chloride hexahydrate CoCl_2_·6H_2_O (99%) was obtained from Shanghai Macklin Biochemical Co., Ltd. Chloroauric acid tetrahydrate (HAuCl_4_·3H_2_O, 99%), sodium borohydride (NaBH_4_, 96%), sodium hydroxide (NaOH, 99.9%), 2-(*n*-morpholino)ethanesulfonic acid (MES, 99%), 1-(3-dimethylaminopropyl)-3-ethylcarbodiimide hydrochloride (EDC·HCl, 98%), *n*-hydroxysuccinimide (NHS, 98%), 4-methylbenzenesulfinic acid (TsOH, 98%), pyruvic acid (PA, 98%), and triethanolamine (TEOA) were acquired from Aladdin Chemistry Co., Ltd. Additionally, 2,4,6-tris(4-formylphenyl)-1,3,5-triazine (TTA) and tris(4-formylphenyl)amine (TFA) were obtained from the Jilin Chinese Academy of Sciences - Yanshen Technology Co., Ltd. Finally, 1,3-dimethyl-2-phenyl-2,3-dihydro-1*H*-benzo[*d*]imidazole (BIH) was purchased from Haohong Biomedical Technology Co., Ltd.

Methods

Fourier transform infrared (FT-IR) spectra were recorded on a Cary 660 spectrometer (Agilent, USA) by using a Deuterated Triglycine Sulfate (DTGS) detector. ^13^C cross-polarization (CP)/magic-angle-spinning (M AS) nuclear magnetic resonance (NMR) spectra were collected on a Bruker AVABCE-III spectrometer with a contact time of 2.0 ms, a recycle delay of 15.0 s, and a sample spinning rate of 6 kHz. ^1^H NMR spectra were recorded on Bruker ACF-400 instruments at 400 MHz. Thermogravimetric (TG) analysis was performed with a heating rate of 10 K min^-1^ under N_2_ atmosphere on a STA409 instrument. CHN elemental analysis was conducted on an elemental analyzer, Vario EL cube. Powder X-ray diffraction (PXRD) patterns were registered on a Rigaku Smart Lab X-ray diffractometer. The scanning microscopy (SEM, Hitachi S-4800) was used to investigate the morphology and the corresponding energy dispersive X-ray spectroscopy (EDX) elemental mapping images, with the operating voltage at 10 kV. High-resolution transmission electron microscopy (HR-TEM) images were recorded on a JEOL JEM-2010 (200 kV) instrument. High-angle annular dark field scanning transmission electron microscope (HAADF-STEM) was carried out on a JEOL GRAND ARM 300CF field-emission transmission electron microscope operated at 200 kV, and the samples were dispersed in ethanol and then dropped onto carbon films (BZ11032a, Beijing Zhongjingkeyi Technology Co., Ltd). A Kratos AXIS Supra X-ray photoelectron spectrometer equipped with Al *K_α_* radiation was used in X-ray photoelectron spectroscopy (XPS) analysis. N_2_ sorption isotherms were measured at 77 K on a BELSORP-Max analyzer. The samples were degassed at 393 K for 3 h to get a vacuum of 10^-3^ Torr before measurement. Desorption was manually stopped when the relative pressure reached *P*/*P_0_*<0.1. The surface areas were calculated based on the Brunauer-Emmett-Teller (BET) equation. The total pore volumes were obtained from the N_2_ sorption isotherm at *P*/*P_0_*=0.99. The pore size distribution was analyzed based on Nonlocal Density Functional Theory (NLDFT), assuming slit pores and a carbon-like adsorbent character. The Co content was determined by inductively coupled plasma (ICP) analysis with a PE Avio200 ICP-OES instrument. UV-vis spectra were measured using a Shimadzu UV-2600 spectrometer, and BaSO_4_ was used as an internal standard. Steady-state photoluminescence (PL) spectra and transient-state PL spectra were measured by RF-5301/PC Spectro-fluorophotometer (Shimadzu, Japan) at room temperature with the excitation wavelength of 420 nm. A ^13^C isotope experiment was conducted by using ^13^CO_2_ (Aldrich, 99.0 atom% for ^13^C), and the gas phase was analyzed by gas chromatography-mass spectrometry (GC-MS, 7890B and 5975C, Agilent Technologies). Transient surface photovoltage (TPV) curves were acquired by a homemade system equipped with a third-harmonic Nd: YAG laser (Polaris II, New Wave Research, USA) and a 500 MHz digital phosphor oscilloscope (Tektronix, USA) Electron paramagnetic resonance (EPR) measurements were carried out on a Bruker model A300 spectrometer.

Photoelectrochemical measurements

Photoelectrochemical measurements were conducted in a standard three-electrode cell at room temperature by using an aqueous Na_2_SO_4_ solution (0.5 M) as the electrolyte. The reference and counter electrodes were Ag/AgCl (KCl saturated) and platinum wire, respectively. For the measurement of photocurrent, the catalyst (3 mg) was mixed with 330 μL of a Nafion ethanol solution (Nafion: ethanol = 1:10), and the mixture was sonicated for 0.5 h. After that, 44 μL of the suspension was dropped on the surface of the glassy carbon electrode to provide the working electrode. Visible light irradiation was provided by a Xe lamp (300 W) with a 400 nm cut-off filter to illuminate the working electrode. Electrochemical impedance spectroscopy (EIS) and Mot-Schottky plots were measured on a PARSTAT MC electrochemical workstation (Princeton, USA) with a similar procedure to photocurrent measurement. During the collection of EIS spectra, the samples were tested with a frequency range from 10 kHz to 0.01 Hz. Mott-Schottky plots were measured at frequencies of 500, 750, and 1000 Hz.

Cyclic voltammetry (CV)

Cyclic voltammetry (CV) was performed in a single-compartment, three-electrode cell containing Ar- or CO_2_-saturated MeCN with 0.10 M TBAPF_6_ as the electrolyte. A 3 mm-diameter glassy carbon disk served as the working electrode, a Pt wire as the counter electrode, and an Ag/AgCl wire as the reference electrode. Potentials are reported versus Ag/AgCl under the stated conditions.

Synthesis of Au_25_ NCs

The Au_25_ NCs were synthesized according to previous work (85,86). Typically, 0.5 mmol of the HAuCl_4_ aqueous solution and 0.75 mmol of the Cys solution were added successively into 200 mL of deionized water under vigorous stirring, followed by adding 30 mL of 1 M NaOH solution. After that, an excessive NaBH_4_ solution (about five times the equivalent of gold) was freshly prepared and added to the above solution, and the mixture was stirred for 3 h. Finally, the obtained products were washed repeatedly with the ethanol/water mixture (V/V = 3:1) and collected after lyophilization. The obtained solids were dissolved in water and defined by UV-visible spectra (fig. S1) and high-resolution electrospray ionization mass spectrometry (ESI-MS, fig. S2), which proved the synthesis of the Au_25_ nanoclusters.

Preparation of Au_25_+TF-COF-COOH-Co

The physically mixed Au_25_+TF-COF-COOH-Co was prepared following the same protocol used for TF-COF-CONH-Au_25_, except that the COF support was replaced with TF-COF-COOH-Co, and the coupling agents NHS, EDC·H_2_O, and MES were omitted.

Determination of apparent quantum efficiency

The apparent quantum efficiency (AQE) was measured under the illumination of a Xe lamp (300 W) with different bandpass filters of 400 ± 10 nm, 420 ± 10 nm, 450 ± 10 nm, 500 ± 10 nm, 550 ± 10 nm, 600 ± 10 nm, and 650 ± 10 nm, respectively. The ILT 950 spectroradiometer was used to measure the intensity of incident monochromatic illumination (15.0 mW cm^-2^). The irradiation area was 3.14 cm^2^. Depending on the amount of CO evolution per hour, AQE was calculated as follows (87):

AQE % = 2 × (*n*_CO or H2_·*N_A_*·*h*·*c*) /(*P*·*S*·*t*·*λ*) × 100% (Eq.1)

Where *N_A_* is Avogadro constant (6.02×10^23^ mol^-1^), *h* is the Planck constant (6.626×10^-34^ J·s), *c* is the speed of light (3×10^8^ m s^-1^), *S* is the irradiation area (cm^2^), *P* is the intensity of irradiation light (W cm^-2^), *t* is the photoreaction time (s), *λ* is the wavelength of the monochromatic light (m).

Calculation of Turnover Number (TON)

The turnover number (TON) was calculated using the following equation (34,88-90):

$TON=\frac{Amounts of products (mol)}{Amounts of active sites in the catalyst (mol)}$ (Eq.2)

Calculation of Turnover Frequency (TOF)

The turnover frequency (TOF) was calculated using the following equation (88-90):

$TOF=\frac{Amounts of products (mol)}{Amounts of active sites in the catalyst \left( mol \right)\times t (h)}$ (Eq.3)

where *t* represents the photoreaction time.

Calculation of CO production selectivity

The selectivity of CO production is evaluated using the following equation (88-90).

$CO selectivity \left( \% \right)=\frac{2\varphi_{CO}}{2\varphi_{CO}+ 2\varphi_{H_{2}}}$ (Eq.4)

Where φ_CO_ represents the mole numbers of generated CO, φ_H2_ represents the mole numbers of generated H_2_.

ESI-MS spectra

The molecular composition of the nanoclusters was characterized using electrospray ionization mass spectrometry (ESI-MS, Q Exactive Orbitrap, Thermo Scientific) (38). The dried Au_25_(Cys)_18_ sample was dissolved in deionized water containing 0.01 µM cesium acetate and introduced into the spectrometer *via* direct infusion at a flow rate of 20 µL·min^-1^. The ESI source was operated in negative-ion mode, with a heated nitrogen drying gas supplied at 5 L·min^-1^. The generated negatively charged ions were subsequently analyzed by the mass spectrometer (MS).

TA spectra

Femtosecond transient absorption (fs-TA) spectra were measured on a pump-probe system (TA100, Ultrafast System) with a maximum time delay of ~8 ns, controlled by a motorized optical delay line. Excitation was provided by a fiber-coupled pump source (1030 nm, 50 kHz), with an excitation wavelength of 343 nm and power of 4.3 mW, with the detection spanning range from 480 to 800 nm.

Quasi *in-situ* XPS analysis

Quasi *in-situ* XPS analysis was performed on a PHI5000VersaProbe IV spectrometer. Samples were pressed into self-supporting wafers, mounted in a polyimide-window microreactor, and purged with He to remove physiosorbed impurities. Spectra were collected first in the dark and then under visible-light irradiation (300 W Xe lamp, λ ≥ 400 nm) using Al Kα excitation.

In-situ ATR-SEIRAS measurements

*In-situ* attenuated total reflection surface enhanced infrared absorption spectroscopy (ATR-SEIRAS) measurement was conducted on a Thermo Nicolet iS50 spectrometer (USA) equipped with a mercury cadmium telluride (MCT) detector. The home-made *in-situ* cell was charged with CO_2_-saturated TEOA/MeCN solution, followed by adding TF-COF-CONH-Au_25_-Co and BIH. After that, the mixture was irradiated with an Xe lamp (300 W), and time-resolved spectra were recorded every 5 min.

Computational detail

All calculations based on density functional theory (DFT) were implemented using the Vienna Ab initio Simulation Package (VASP). We have employed the first principles (91,92) to perform DFT calculations within the generalized gradient approximation (GGA) using the Perdew-Burke-Ernzerhof (PBE) formulation (93). We have chosen the projected augmented wave (PAW) potentials (94,95) to describe the ionic cores and taken valence electrons into account using a plane wave basis set with a kinetic energy cutoff of 520 eV. The Brillouin zone was sampled with a 3 × 3 × 1 Gamma mesh. Electronic self-consistency was deemed converged when the total-energy change was below 10^-6^ eV, and atomic positions were relaxed until the residual forces fell below 0.02 eV Å^-1^. Slab models contained 15 Å of vacuum along z to suppress spurious interactions. To mimic the MeCN/TEOA reaction medium, implicit solvation was applied via VASPsol with ε = 40.0 and surface-tension parameter τ = 0.0055 (96-98).

Periodic slabs were built from experimental guided COF frameworks for TF-COF-Co, TF-COF-COOH-Co, and TF-COF-CONH-Au_25_-Co. In the Au-containing model, an atomically precise Au_25_ cluster was covalently tethered to the ‒CO‒NH‒ linker *via* the amide coupling inferred experimentally (99,100). To control computational cost while retaining interfacial chemistry, the thiolate ligands that bond to the COF were kept explicitly, whereas distal ligand sections were truncated after validation. The cobalt site was modeled as an electrostatically immobilized [Co(bpy)_3_]^2+^ (resting state). For catalysis, the photo-reduced [Co(bpy)_2_]^2+^ motif, identified as the competent CO_2_-reduction species, was constructed and used to evaluate reaction energetics (fig. S77) (55,56).

According to the *in-situ* ATR-SEIRAS and previous mechanistic study, the free energy profiles for CO_2_ reduction to CO were calculated according to the following pathway (101):

CO_2_ + * → *CO_2_ (Eq.5)

*CO_2_ + H^+^ + e^-^ → *COOH (Eq.6)

*COOH + H^+^ + e^-^ → *CO + H_2_O (Eq.7)

*CO → CO + * (Eq.8)

where* stands for active centers during the photocatalytic CO_2_ reduction process.

The free energy of the adsorbed intermediates *CO_2_, *COOH, and *CO and non-adsorbed gas molecules was calculated using the following equation (101):

$G=E_{elec}+E_{ZPE}+\int C_{P}dT-TS$ (Eq.9)

where *E_elec_* is the electronic energy calculated by DFT; *E_ZPE_* is the zero-point energy (ZPE), *C_P_* is heat capacity, *T* is temperature, and *S* is entropy. The changes in free energy for each step were calculated. At the standard hydrogen electrode (SHE), the free energy of (H^+^ + e^‑^) was replaced with the 1/2 H_2_(g).

Fig. S1.

UV-vis spectrum of Au_25_ NCs.

Fig. S2.

ESI-mass spectra of Au_25_(Cys)_18_ from (**A**) 1500 ~ 4000 and (**B**) 2350 ~ 2410. The inset in B is the observed isotope pattern in #1. In the m/z 1500 ~ 4000 window, the most intense envelope appeared at *m/z*  = 2361.45 (fig. S2A). The expanded view (fig. S2B) revealed a primary peak (Peak #1) with peak-to-peak spacing of ~0.333 *m/z*, consistent with a 3-charge state and assignable to [Au_25_(Cys)_18_-3H]^3-^. Deconvolution gave a neutral mass of 7084.35 Da, in excellent agreement with the theoretical value of Au_25_(Cys)_18_. The adjacent minor peaks (Peaks #2 ~ #7) formed a regular Na^+^-adduct series: (#2) [Au_25_(Cys)_18_-4H^+^+Na]^3-^ (MW 7106.31 Da), (#3) [Au_25_(Cys)_18_-5H^+^+2Na]^3-^ (7128.30 Da), (#4) [Au_25_(Cys)_18_-6H^+^+3Na]^3-^ (7150.26 Da), (#5) [Au_25_(Cys)_18_-7H^+^+4Na]^3-^ (7172.25 Da), (#6) [Au_25_(Cys)_18_-8H^+^+5Na]^3-^ (7194.24 Da), and (#7) [Au_25_(Cys)_18_-9H^+^+6Na]^3-^ (7216.23 Da), which is the expected fingerprint of a single parent stoichiometry under soft ESI conditions. Across a broader m/z scan, we resolve three additional charge-state envelopes at m/z = 1808.09 (2^-^), 2411.77 (3^-^), and 3542.63 (4^-^), each matching the calculated isotopic distributions for Au_25_(Cys)_18_. Crucially, no detectable signals corresponding to alternative nuclearities Au*_n_*(SR)*_m_* (*n* ≠ 25) were observed in the spectrum. Together, the charge-state series, isotopic fits, and systematic Na^+^ adduction unambiguously confirm that the product is a single, atomically precise Au_25_(Cys)_18_ nanoclusters.


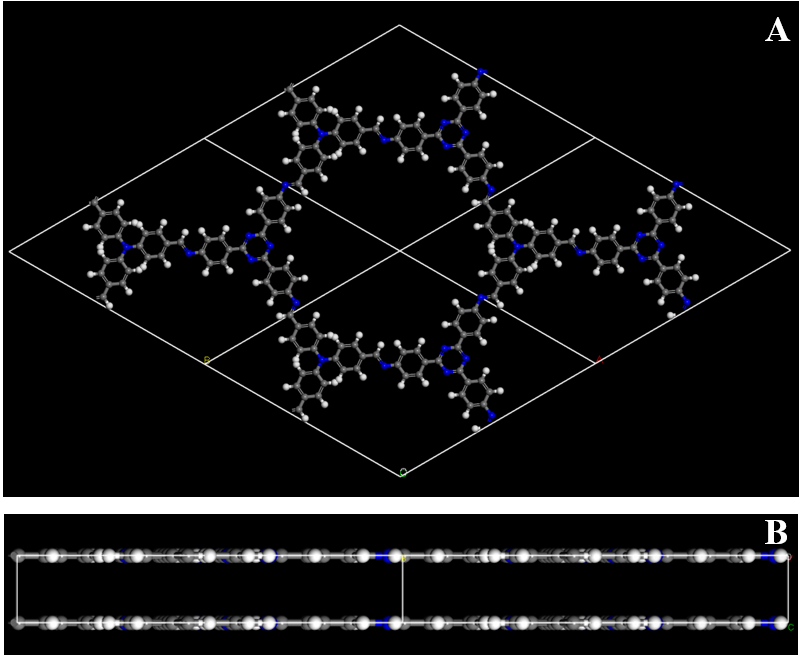


Fig. S3.

The computationally determined structures of TF-COF. (**A**) Top view and (**B**) side view of the theoretical structure of TF-COF with eclipsed (AA) stacking arrangement.


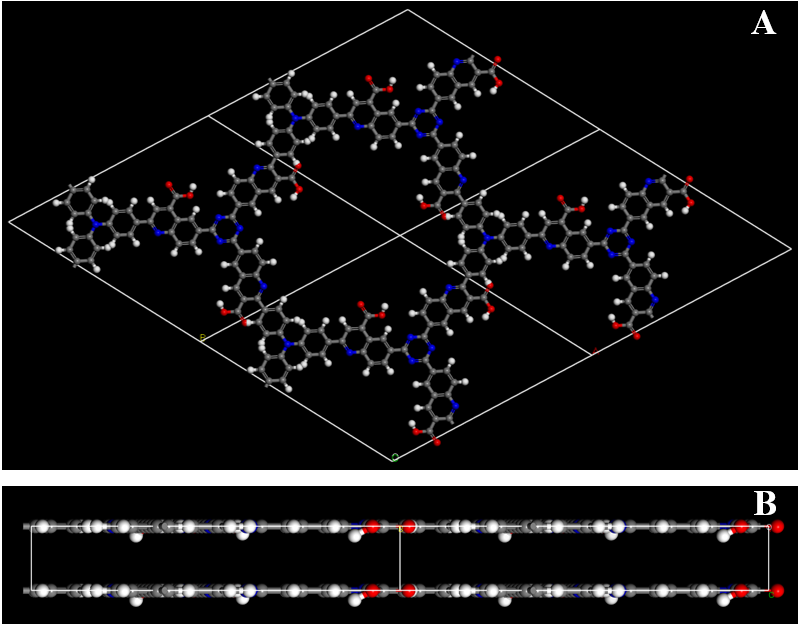


Fig. S4.

The computationally determined structures of TF-COF-COOH. (**A**) Top view and (**B**) side view of the theoretical structure of TF-COF-COOH with eclipsed (AA) stacking arrangement.

Fig. S5.

PXRD patterns of TF-COF after treatment in 6 M HCl, 3 M NaOH, 1 M NaBH_4_, and 0.5 M H_2_O_2_ for 1 day.

Fig. S6.

PXRD patterns of TF-COF-COOH and TF-COF-CONH-Au_25_.

Fig. S7.

(**A**-**B**) FT-IR spectra of TF-COF, TF-COF-COOF, and TF-COF-CONH-Au_25_.

Fig. S8.

The ^13^C NMR spectrum of TF-COF.

Fig. S9.

The ^13^C NMR spectrum of TF-COF-COOH.

Fig. S10.

The ^13^C NMR spectrum of TF-COF-CONH-Au_25_.

Fig. S11.

(**A**) N_2_ sorption isotherms, and (**B**) pore size distribution curves of TF-COF, TF-COF-COOH, and TF-COF-CONH-Au_25_.

Fig. S12.

CO_2_ adsorption isotherms of TF-COF, TF-COF-COOH, and TF-COF-CONH-Au_25_ at 298 K.


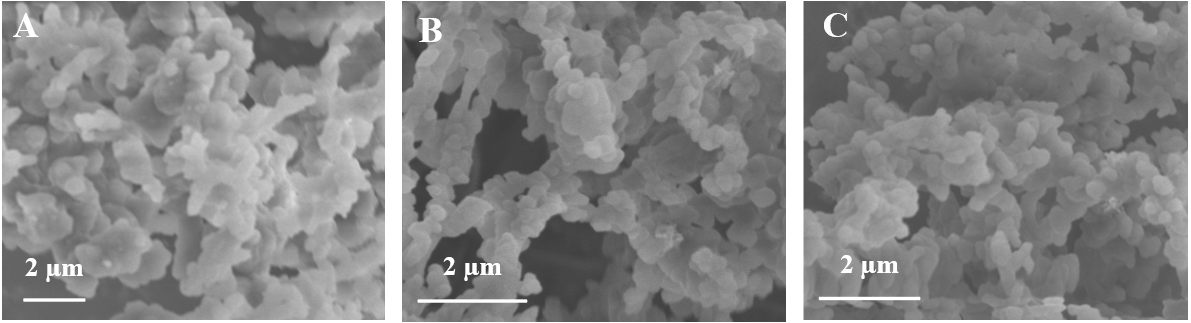


Fig. S13.

SEM images of (**A**) TF-COF, (**B**) TF-COF-COOH, and (**C**) TF-COF-CONH-Au_25_.


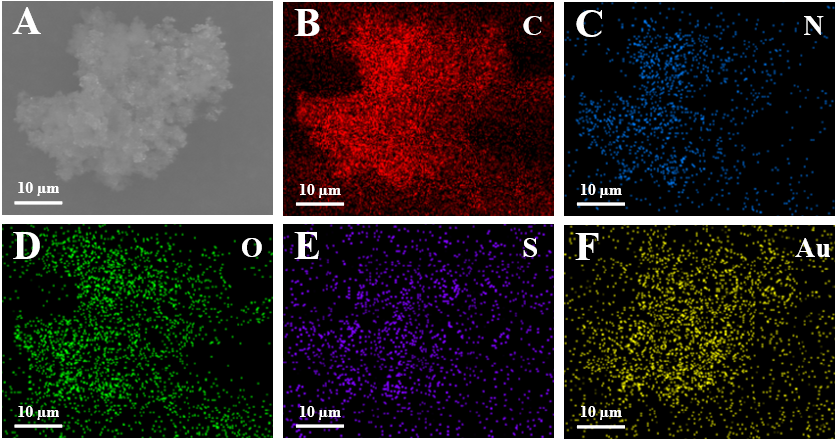


Fig. S14.

SEM image and elemental mapping images of TF-COF-CONH-Au_25_ showing the uniform presence of C, N, O, S, and Au.


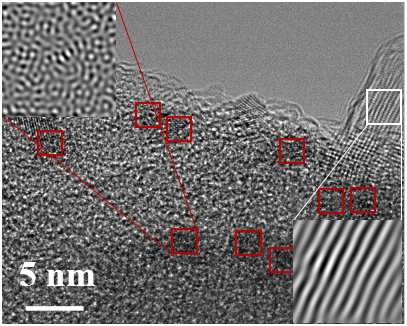


Fig. S15.

HR-TEM image of TF-COF-CONH-Au_25_. The observed interplanar spacings of approximately 0.38 nm came from the (001) crystal plane of TF-COF-COOH, consistent with the low-angle reflection in the PXRD pattern. The fringes near 0.235 nm (Au(111)) or 0.204 nm (Au(200)) that could be a diagnostic of aggregated metallic Au nanoparticles were not observed.

Fig. S16.

Survey scan XPS spectra of TF-COF, TF-COF-COOH, and TF-COF-CONH-Au_25_.

Fig. S17.

High-resolution N 1s XPS spectra of TF-COF, TF-COF-COOH, and TF-COF-CONH-Au_25_.

Fig. S18.

High-resolution C 1s XPS spectra of TF-COF, TF-COF-COOH, and TF-COF-CONH-Au_25_.

Fig. S19.

High-resolution O 1s XPS spectra of TF-COF-COOH and TF-COF-CONH-Au_25_.

Fig. S20.

High-resolution (**A**) Au 4f and (**B**) S 2p XPS spectra of TF-COF-CONH-Au_25_ and Au_25_ NCs.

Fig. S21.

FT-EXAFS fitting curves in the R space of TF-COF-CONH-Au_25_.

Fig. S22.

FT-EXAFS fitting curves in the R space of Au foil.

Fig. S23.

FT-EXAFS spectra at the Au *L*_3_-edge in the *k* space of TF-COF-CONH-Au_25_-Co, TF-COF-CONH-Au_25_, and Au foil.

Fig. S24.

Neutron PDF and the corresponding differential profiles for TF-COF-COOH and TF-COF-CONH-Au_25_.

Fig. S25.

Neutron *d*-PDF, Rietveld refined profile, and their difference obtained by subtracting the PDF data of TF-COF-CONH-Au_25_ from TF-COF-COOH. The blue point, red line, and orange line indicate the measured data, calculated data, and residual curves, respectively.

Fig. S26.

Simulated neutron PDF profiles of Au–Au, Au–S, C–S, and C–O/C=O bonds in Au_25_ NCs.

Fig. S27.

Zeta potential distribution of TF-COF-CONH-Au_25_, TF-COF-COOH, TF-COF, and [Co(bpy)_3_]^2+^.

Fig. S28.

PXRD patterns of TF-COF-Co, TF-COF-COOF-Co, and TF-COF-CONH-Au_25_-Co.

Fig. S29.

FT-IR spectra of TF-COF-Co, TF-COF-COOF-Co, and TF-COF-CONH-Au_25_-Co.

Fig. S30.

(**A**) N_2_ sorption isotherms, and (**B**) pore size distribution curves of TF-COF-Co, TF-COF-COOH-Co, and TF-COF-CONH-Au_25_-Co.


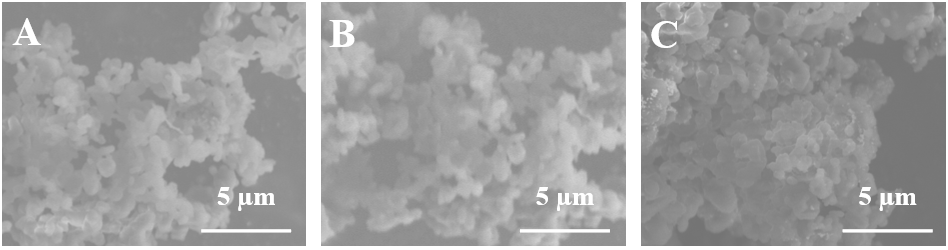


Fig. S31.

SEM images of (**A**) TF-COF-Co, (**B**) TF-COF-COOH-Co, and (**C**) TF-COF-CONH-Au_25_-Co.


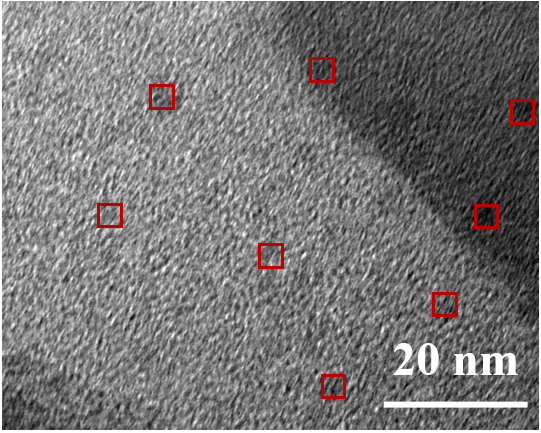


Fig. S32.

HR-TEM image of TF-COF-CONH-Au_25_-Co.

Fig. S33.

Survey scan XPS spectra of TF-COF-Co, TF-COF-COOH-Co, and TF-COF-CONH-Au_25_-Co.

Fig. S34.

High-resolution Au 4f XPS spectra of TF-COF-CONH-Au_25_ and TF-COF-CONH-Au_25_-Co.

Fig. S35.

High-resolution Co 2p XPS spectra of TF-COF-Co, TF-COF-COOH-Co, and TF-COF-CONH-Au_25_-Co.

Fig. S36.

O 1s XPS spectra of TF-COF-COOH and TF-COF-COOH-Co.

Fig. S37.

High-resolution O 1s XPS spectra of TF-COF-CONH-Au_25_ and TF-COF-CONH-Au_25_-Co.

Fig. S38.

FT-EXAFS fitting curves in the R space of TF-COF-COOH-Co.

Fig. S39.

FT-EXAFS fitting curves in the R space of TF-COF-Co.

Fig. S40.

FT-EXAFS fitting curves in the R space of Co foil.

Fig. S41.

FT-EXAFS fitting curves in the R space of CoO.

Fig. S42.

FT-EXAFS spectra at the Co *K*-edge in the *k* space of TF-COF-CONH-Au_25_-Co, TF-COF-COOH-Co, TF-COF-Co, Co foil, and CoO.

Fig. S43.

Neutron PDF and the corresponding differential profiles for TF-COF-CONH-Au_25_ and TF-COF-CONH-Au_25_-Co.

Fig. S44.

Neutron *d*-PDF, Rietveld refined profile, and their difference obtained by subtracting the PDF data of TF-COF-CONH-Au_25_-Co from TF-COF-CONH-Au_25_. The blue point, red line, and orange line indicate the measured data, calculated data, and residual curves, respectively.

Fig. S45.

Simulated neutron PDF profiles of Co–N, C–C, and C–N bonds in [Co(bpy)_3_]^2+^.

Fig. S46.

Time-resolved generation of gas (CO and H_2_) over TF-COF-CONH-Au_25_-Co. Reaction conditions: catalyst (1 mg), BIH (20 mg), solvent (4 mL), TEOA (1 mL), CO_2_ (1 atm), visible light irradiation (λ ≥ 400 nm), RT.

Fig. S47.

Quasi *in-situ* diffuse reflectance UV-visible spectra of TF-COF-CONH-Au_25_-Co in MeCN/BIH under visible-light irradiation (**A**) with TEOA and (**B**) without TEOA.

Fig. S48.

Time-resolved generation of gas (CO and H_2_) over TF-COF-COOH-Co in 24 h. Reaction conditions: catalyst (1 mg), BIH (20 mg), solvent (4 mL), TEOA (1 mL), CO_2_ (1 atm), visible light irradiation (λ ≥ 400 nm), RT.

Fig. S49.

Time-resolved generation of gas (CO and H_2_) over TF-COF-Co in 24 h. Reaction conditions: catalyst (1 mg), BIH (20 mg), solvent (4 mL), TEOA (1 mL), CO_2_ (1 atm), visible light irradiation (λ ≥ 400 nm), RT.

Fig. S50.

Photocatalytic CO_2_ reduction over Co-free catalysts. Reaction conditions: catalyst (1 mg), BIH (20 mg), solvent (4 mL), TEOA (1 mL), CO_2_ (1 atm), visible light irradiation (λ ≥ 400 nm), RT, 6 h.

Fig. S51.

Photocatalytic CO_2_ reduction over different catalysts. Reaction conditions: BIH (20 mg), solvent (4 mL), TEOA (1 mL), CO_2_ (1 atm), visible light irradiation (λ ≥ 400 nm), RT, 6 h.

Fig. S52.

AQY over TF-COF-CONH-Au_25_-Co and comparison along with the absorption of TF-COF-CONH-Au_25_-Co.

Fig. S53.

Gas (CO and H_2_) production rates of TF-COF-CONH-Au_25_-Co under various reaction conditions.

Fig. S54.

^1^H NMR spectrum of the liquid phase from the reaction system after photocatalytic CO_2_ reduction over TF-COF-CONH-Au_25_-Co. Reaction conditions: catalyst (1 mg), BIH (20 mg), MeCN (4 mL), TEOA (1 mL), CO_2_ (1 atm), visible light irradiation (λ ≥ 400 nm), RT, 6 h.

Fig. S55.

PXRD patterns of TF-COF-CONH-Au_25_-Co before and after the photocatalytic test.

Fig. S56.

High-resolution (**A**) Au 4f and (**B**) Co 2p XPS spectra of recovered TF-COF-CONH-Au_25_-Co after photocatalytic test.

Fig. S57.

Recyclability of physically mixed Au_25_+TF-COF-COOH-Co.

Fig. S58.

Tauc plots and band gaps of TF-COF (light blue), TF-COF-COOH (blue), and TF-COF-CONH-Au_25_ (red).

Fig. S59.

(**A**) UV–vis spectra, (**B**) tauc plots and band gaps of TF-COF-Co (light blue), TF-COF-COOH-Co (blue), and TF-COF-CONH-Au_25_-Co (red).

Fig. S60.

Mott-Schottky plots of (**A**) TF-COF and (**B**) TF-COF-Co.

Fig. S61.

Mott-Schottky plots of (**A**) TF-COF-COOH and (**B**) TF-COF-COOH-Co.

Fig. S62.

Mott-Schottky plots of (**A**) TF-COF-CONH-Au_25_ and (**B**) TF-COF-CONH-Au_25_-Co.

Fig. S63.

Band gap structures of (**A**) COF and (**B**) COF-Co.

Fig. S64.

EIS Nyquist plots of COF and COF-Co.

Fig. S65.

Transient photocurrent density under Xenon lamp (≥ 400 nm) irradiation.

Fig. S66.

(**A**) Steady-state PL spectra, and (**B**) time-resolved PL decay spectra of TF-COF and TF-COF-Co.

Fig. S67.

(**A**) Steady-state PL spectra, and (**B**) time-resolved PL decay spectra of TF-COF-COOH and TF-COF-COOH-Co.

Fig. S68.

(**A**) Steady-state PL spectra, and (**B**) time-resolved PL decay spectra of TF-COF-CONH-Au_25_ and TF-COF-CONH-Au_25_-Co.

Fig. S69.

TPV relaxation curves of TF-COF-Co, TF-COF-COOH-Co, and TF-COF-CONH-Au_25_-Co.

Fig. S70.

(**A**) Maximum charge extraction time (T_max_) and amount of charge extraction (A), (**B**) attenuation constants (τ) in TPV relaxation curves of TF-COF-Co, TF-COF-COOH-Co, and TF-COF-CONH-Au_25_-Co.

Fig. S71.

(A) 2D mapping TA spectra and (B) Fs-TA spectra at different decay times of TF-COF-CONH-Au_25_.

Fig. S72.

(A) 2D mapping TA spectra and (B) Fs-TA spectra at different decay times of TF-COF-COOH.

Fig. S73.

(A) 2D mapping TA spectra and (B) Fs-TA spectra at different decay times of TF-COF.

Fig. S74.

Representative kinetic traces along with their global fitting results of the fs-TA spectra of TF-COF-CONH-Au_25_.

Fig. S75.

Representative kinetic traces along with their global fitting results of the fs-TA spectra of TF-COF-COOH.

Fig. S76.

Representative kinetic traces along with their global fitting results of the fs-TA spectra of TF-COF.

Fig. S77.

CVs of [Co(bpy)_3_]^2+^ (0.4 mM) in anhydrous TBAPF_6_/MeCN (0.1 M) solution at V = 0.1 V s^-1^ under Ar and CO_2_ atmosphere.

Fig. S78.

*In-situ* ATR-SEIRAS for the adsorption and photocatalytic conversion of CO_2_ on TF-COF-CONH-Au_25_.

Fig. S79.

*In-situ* ATR-SEIRAS for the adsorption and photocatalytic conversion of CO_2_ on TF-COF-COOH-Co.


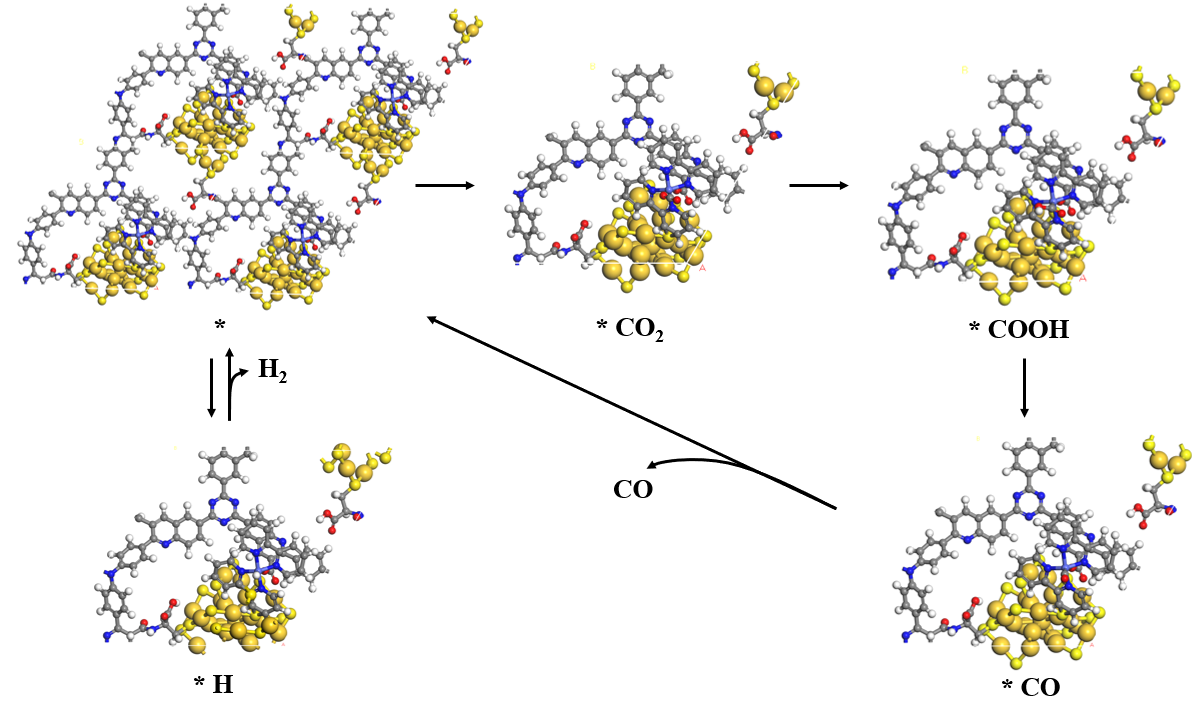


Fig. S80.

Possible mechanism of the CO_2_ reduction and H_2_ evolution catalyzed by TF-COF-CONH-Au_25_-Co.


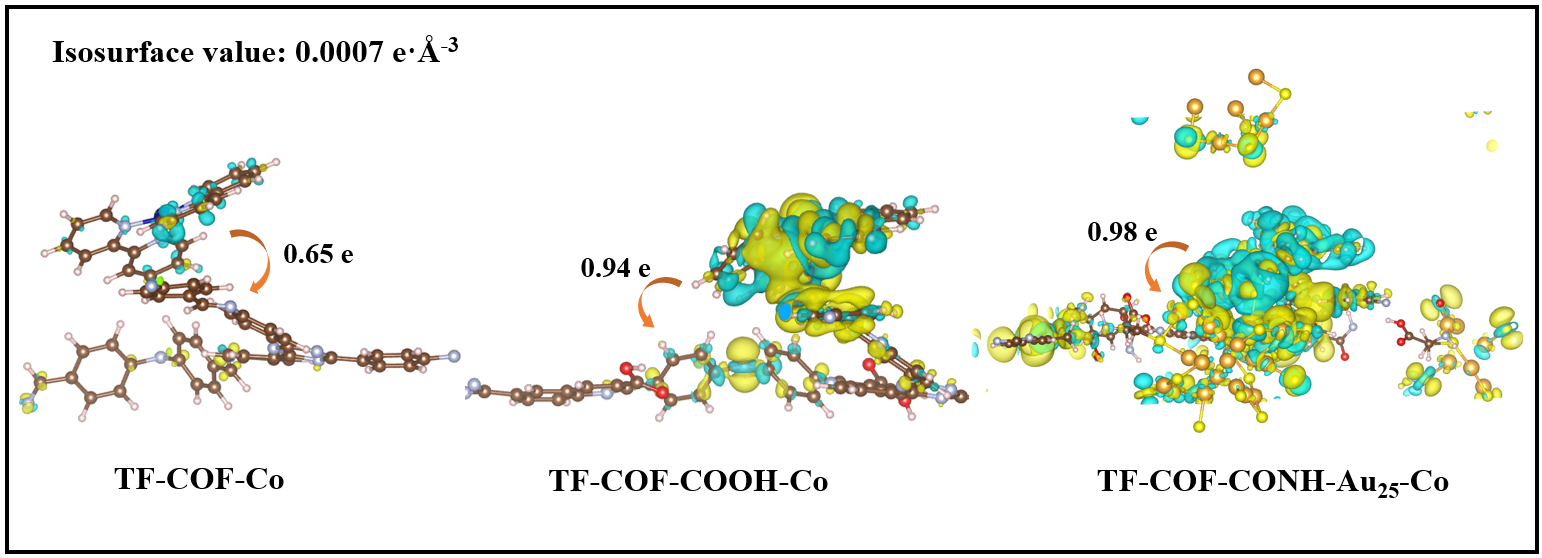


Fig. S81.

Charge density redistribution (charge transfer) and Bader charge results of TF-COF-Co, TF-COF-COOH-Co, and TF-COF-CONH-Au_25_-Co. Yellow and blue isosurfaces are represented as electron accumulation and depletion, respectively.

Fig. S82.

Correlation between Co 2p XPS peak shifts (ΔBE) and DFT-derived Bader charges (Δq). Linear regression analysis of the three data points yielded the following relationship: ΔBE (eV) = 1.20 * Δq (e) - 0.005 (R^2^ ≈ 0.95).

Table S1.

Textual properties of COF and COF-Co

| sample | S_BET_*^a^* (m^2^ g^-1^) | V_p_^b^ (cm^3^ g^-1^) | D_av_*^c^* (nm) |
| --- | --- | --- | --- |
| TF-COF | 1510 | 1.17 | 2.13 |
| TF-COF-COOH | 1215 | 0.90 | 2.13 |
| TF-COF-CONH-Au_25_ | 640 | 0.48 | 1.71 |
| TF-COF-Co | 1243 | 0.95 | 2.13 |
| TF-COF-COOH-Co | 1051 | 0.62 | 2.13 |
| TF-COF-CONH-Au_25_-Co | 401. | 0.32 | 1.70 |

*^a^*BET surface area. *^b^*Total pore volume. *^c^*Average pore size.

Table S2.

EXAFS fitting parameters at the Au *L*_3_-edge

| sample | Shell | CN | σ^2^ (×10^-3^ Å^2^) | ΔE_0_ (eV) | R (Å) | R-*factor* |
| --- | --- | --- | --- | --- | --- | --- |
| Au foil | Au–Au | 12* | 6.3 | 3.53 | 2.8 | 0.007 |
| TF-COF-CONH-Au_25_ | Au–S | 2.1 | 8.6 | 8.39 | 2.3 | 0.011 |
|  | Au–Au_1_ | 1.5 | 9.5 |  | 2.8 |  |
|  | Au–Au_2_ | 1.6 | 9.7 |  | 3.1 |  |
|  | Au–Au_3_ | 1.9 | 5.6 |  | 3.3 |  |
| TF-COF-CONH-Au_25_-Co | Au–S | 1.5 | 7.4 | 9.4 | 2.3 | 0.020 |
|  | Au–Au_1_ | 1.4 | 7.1 |  | 2.9 |  |
|  | Au–Au_2_ | 1.5 | 9.8 |  | 3.1 |  |
|  | Au–Au_3_ | 1.8 | 4.5 |  | 3.3 |  |

CN: coordination number, R: interatomic distance (the bond length between central atoms and surrounding coordination atoms) based on fitting results, ΔE_0_: inner potential correction, σ^2^: Debye-Waller factor, and the R-*factor* is used to value the goodness of fit. Error bounds that characterize the structural parameters obtained by EXAFS fitting were estimated as CN ± 20%, R ± 1%, *σ*^2^ ± 20%, and Δ*E*_0_ ± 20%.

Table S3.

EXAFS fitting parameters at the Co *K*-edge

| sample | Shell | CN | σ^2^ (×10^-3^ Å^2^) | ΔE_0_ (eV) | R (Å) | R-*factor* |
| --- | --- | --- | --- | --- | --- | --- |
| Co foil | Co–Co | 12* | 5.3 | 4.3 | 2.5 | 0.001 |
| CoO | Co–O | 6.8 | 1.7 | 2.8 | 2.1 | 0.017 |
|  | Co–Co | 12.1 | 1.0 |  | 3.0 |  |
| TF-COF-CONH-Au_25_-Co | Co–N | 5.5 | 6.1 | 4.8 | 2.1 | 0.033 |
| TF-COF-COOH-Co | Co–N | 5.6 | 12.0 | 5.8 | 2.1 | 0.017 |
| TF-COF-Co | Co–N | 6.8 | 5.5 | 6.3 | 2.1 | 0.011 |

CN: coordination number, R: interatomic distance (the bond length between central atoms and surrounding coordination atoms) based on fitting results, ΔE_0_: inner potential correction, σ^2^: Debye-Waller factor, and the R-*factor* is used to value the goodness of fit. Error bounds that characterize the structural parameters obtained by EXAFS fitting were estimated as CN ± 20%, R ± 1%, *σ*^2^ ± 20%, and Δ*E*_0_ ± 20%.

Table S4.

Comparison of visible-light-driven CO_2_ reduction to CO

| Cat. | Co-cat. | amount | Sacrificial agent | Irradiation condition |  | Reaction time | Product rate  (μmol g^−1^ h^−1^) | TON | TOF (h^-1^) | Ref. |
| --- | --- | --- | --- | --- | --- | --- | --- | --- | --- | --- |
| **Heterogeneous photocatalysts** | | | | | | | | | | |
| TF-COF-CONH-Au_25_-Co | / | 1 mg | TEOA/BIH | λ>400 nm  (300 W Xe lamp), 400 mW cm^-2^ | Pyrex cell | 24 h | 2321.9 | 171.9 | 7.2 | This work |
| Cu–COF | / | 5 mg | TEOA | λ>420 nm  (300 W Xe lamp) | Pyrex cell | 5 h | 206 | N/A | N/A | (102) |
| LaNi-Phen/COF-5 | / | 10 mg | BIH | (300 W Xe lamp), 100 mW cm^-2^ | Pyrex glass reaction cell | 5 h | 605.8 | N/A | N/A | (52) |
| pNJU-319Fe | / | 10 mg | TEOA | λ>400 nm  (300 W Xe lamp) | / | 10 h | 68.8 | N/A | N/A | (103) |
| COF-RuBpy-Co | / | 5 mg | TEOA/BIH | λ>420 nm  (300 W Xe lamp) | / | 20 h | 547 | 18 | 0.9 | (104) |
| 1D PyTTA-COF | / | 3 mg | BIH | λ>420 nm  (Xe lamp) | / | 8 h | 125.4 | 2.2 | 0.3 | (29) |
| CdS@COF | / | 5 mg | BIH | λ>420 nm  (300 W Xe lamp) | / | 8 h | 507.1 | N/A | N/A | (105) |
| sp2c-COFdpy-Co | / | 20 mg | TEOA | λ>420 nm  (300 W Xe lamp) | Pyrex glass cell | 6 h | 1000 | N/A | N/A | (59) |
| Re-COF | / | 0.9 mg | TEOA | λ>420 nm  (225 W Xe lamp) | 11 mL septum-sealed glass vials | 20 h | 750 | 48 | 2.4 | (61) |
| MCOF-Ru/Re | / | 2 mg | TEOA/BIH | λ>420 nm  (300 W Xe lamp) | 25 mL quartz reaction vessel | 5 h | 1840 | 29 | 5.8 | (106) |
| PY-CN-BIP-Ni | / | 5 mg | BIH | λ>420 nm  (300 W Xe lamp) | Pyrex reactor | 5 h | 553.3 | 4.24 | 4.24 | (107) |
| ZnZn-Salen-COF | / | 10 mg | H_2_O | λ>420 nm  (300 W Xe lamp) | 100 mL Pyrex reactor | 6 h | 150.9 | N/A | N/A | (108) |
| **Homogeneous photocatalysts*** | | | | | | | | | | |
| COOH-COF | [Co(bpy)_3_]^2+^ | 10 mg | TEA | Visible light  Xe lamp (300 W) | / | 3 h | 3690 | 55.4 | 18.5 | (55) |

Table S4.

Continued

| Catalyst | Co-catalyst |  | Sacrificial agent | Irradiation condition |  | Reactor time | Product rate  (μmol g^−1^ h^−1^) | TON | TOF (h^-1^) | Ref. |
| --- | --- | --- | --- | --- | --- | --- | --- | --- | --- | --- |
| CN/CTF | [Co(bpy)_3_]^2+^ | 5 mg | TEOA | Xe lamp (300 W), 200 mW cm^-2^ | Gas phase reaction | 3 h | 151.1 | 15.1 | 0.5 | (109) |
| TMBen-Pyrene | [Ni(bpy)_3_]^2+^ | 10 mg | TEOA/BIH | λ>420 nm  (300 W Xe lamp) | A 25 mL tube | 5 h | 93 | 1.6 | 0.3 | (62) |
| g-C_3_N_4_ (NH)/COF | [Co(bpy)_3_]^2+^ | 20 mg | TEOA | λ>400 nm  (300 W Xe lamp) | / | 7 h | 562.5 | 78.8 | 11.3 | (110) |
| FBP-COF | [Co(bpy)_3_]^2+^ | 2 mg | TEOA | λ>420 nm  (300 W Xe lamp), 550 mW cm^-2^ | A quartz flask | 30 h | 2080 | 62.4 | 2.1 | (111) |
| Co-Por-BDT | Re(bpy)(CO)_3_Cl | 5 mg | TEOA | λ>420 nm  (300 W Xe lamp) | 20 mL vial | 6 h | 1424 | N/A | N/A | (112) |
| PI-COF-TT | [Ni(bpy)_3_]^2+^ | 10 mg | TEOA | λ>420 nm  (300 W Xe lamp), 780 mW cm^-2^, 313 K | / | 4 h | 483.25 | 3.5 | 0.9 | (75) |

Homogeneous photocatalysts*: In photocatalytic systems, the metal active centers are dispersed in the solvent in a homogeneous condition.

TEOA: triethanolamine, TEA: triethanolamine, BIH: 1,3-dimethyl-2-phenylbenzimidazoline, bpy: 2,2’-bipyridine, N/A: not available.

Table S5.

Fitted TRPL parameters

| Sample | *τ*_1_ (ns) | A_1_ (%) | *τ*_2_ (ns) | A_2_ (%) | *τ*_ave_ (ns) |
| --- | --- | --- | --- | --- | --- |
| TF-COF | 0.64 | 91.51 | 8.29 | 8.49 | 1.29 |
| TF-COF-COOH | 0.8 | 86.92 | 5.55 | 13.08 | 1.42 |
| TF-COF-CONH-Au_25_ | 0.93 | 93.15 | 8.44 | 6.85 | 1.44 |
| TF-COF-Co | 0.67 | 92.74 | 9.89 | 7.26 | 1.34 |
| TF-COF-COOH-Co | 0.84 | 90.62 | 7.28 | 9.38 | 1.45 |
| TF-COF-CONH-Au_25_-Co | 0.86 | 87.43 | 7.37 | 12.57 | 1.68 |

The lifetime fitted by the following triexponential equation: y = A_1_exp(-*t*/τ_1_) + A_2_exp(-*t*/τ_2_) + y_0_. The average lifetime is obtained by the following equation: τ_ave_ =(A_1_τ_12_ + A_2_τ_22_)/(A_1_τ_1_ + A_2_τ_2_).

Table S6.

Fitted TPV parameters

| Sample | A | T_max_ | *τ* (ms) |
| --- | --- | --- | --- |
| TF-COF | 0.0033 | 0.0300 | 0.104 |
| TF-COF-COOH | 0.0049 | 0.0178 | 0.117 |
| TF-COF-CONH-Au_25_ | 0.0058 | 0.0125 | 0.124 |
| TF-COF-Co | 0.0066 | 0.0238 | 0.121 |
| TF-COF-COOH-Co | 0.0074 | 0.0115 | 0.132 |
| TF-COF-CONH-Au_25_-Co | 0.0104 | 0.0113 | 0.140 |

Integral area (A): the capability of the catalysts to produce photoinduced electrons. T_max_ values: the time required for the intensity to reach the maximum. Attenuation constants (τ): fitting of the normalized TPV decay curves.

Table S7.

Time components deduced from global fitting of TAS

| Sample | *τ*_1_ (ps) | *τ*_2_ (ps) | *τ*_3_ (ps) |
| --- | --- | --- | --- |
| TF-COF | 3.99 | 365.2 | / |
| TF-COF-COOH | 4.89 | 437.6 | / |
| TF-COF-CONH-Au_25_ | 5.87 | 518.9 | / |
| TF-COF-CONH-Au_25_-Co | 0.93 | 34.4 | 982.3 |
